# Social complexity, modernity and suicide: an assessment of Durkheim’s suicide from the perspective of a non-linear analysis of complex social systems

**DOI:** 10.1186/s40064-016-1799-z

**Published:** 2016-03-25

**Authors:** Rosalia Condorelli

**Affiliations:** Department of Political and Social Sciences, Catania University, 8 Vittorio Emanuele II, 95131 Catania, Italy

**Keywords:** Modernization and suicide, Social adaptation process, Dissipative structures, Complex adaptive social systems, Social emergence, Nonlinear social interaction system, Emergentist social change process

## Abstract

Can we share even today the same vision of modernity which Durkheim left us by its suicide analysis? or can society ‘surprise us’? The answer to these questions can be inspired by several studies which found that beginning the second half of the twentieth century suicides in western countries more industrialized and modernized do not increase in a constant, linear way as modernization and social fragmentation process increases, as well as Durkheim’s theory seems to lead us to predict. Despite continued modernizing process, they found stabilizing or falling overall suicide rate trends. Therefore, a gradual process of adaptation to the stress of modernization associated to low social integration levels seems to be activated in modern society. Assuming this perspective, the paper highlights as this tendency may be understood in the light of the new concept of social systems as complex adaptive systems, systems which are able to adapt to environmental perturbations and generate as a whole surprising, emergent effects due to nonlinear interactions among their components. So, in the frame of Nonlinear Dynamical System Modeling, we formalize the logic of suicide decision-making process responsible for changes at aggregate level in suicide growth rates by a nonlinear differential equation structured in a logistic way, and in so doing we attempt to capture the mechanism underlying the change process in suicide growth rate and to test the hypothesis that system’s dynamics exhibits a restrained increase process as expression of an adaptation process to the liquidity of social ties in modern society. In particular, a Nonlinear Logistic Map is applied to suicide data in a modern society such as the Italian one from 1875 to 2010. The analytic results, seeming to confirm the idea of the activation of an adaptation process to the liquidity of social ties, constitutes an opportunity for a more general reflection on the current configuration of modern society, by relating the Durkheimian Theory with the Halbwachs’ Theory and most current visions of modernity such as the Baumanian one. Complexity completes the interpretative framework by rooting the generating mechanism of adaptation process in the precondition of a new General Theory of Systems making the non linearity property of social system’s interactions and surprise the functioning and evolution rule of social systems.

## Introduction

Current sociological research supports the idea according to which *Egoistic suicide* is the distinctive product of modernity, showing Durkheim’s acquisitions still valid today. Suicide proves to be the tangible sign of that modernization process that, on one hand, while it contracts the sphere of existence under the authority of traditions and leads toward autonomy, toward personal responsibility and individualism which in itself is desirable, on the other hand, it simultaneously nurses the germs of social malaise identifying its most dangerous manifestations in group disintegration, weakening of primary ties and social isolation. The peculiar aspect of Durkheim lies in having depicted with efficaciousness the dark side of *freedom*. If it is true that the relentless progress of individualism frees man from tradition’s shackles, it is likewise true that freedom comes at a price, and the price is isolation and even more: paradoxically, it is the loss of one’s identity, the loss of life’s meaning itself or of every reason of existing. Durkheim wrote vivid pages on this aspect of modernity, on the existential void which represents the so called *crises of modern man*. More specifically, life no longer has any sense because it has no purpose and it has no purpose simply because society—the family, the Church, the Fatherland—have become more and more extraneous to the individual. On one hand, man can no longer do without living according to himself and to his dictates, but, on the other hand, he cannot avoid the thought that efforts of every his activity will end in nothingness since there is no longer anything to which they are directed. In short, for Durkheim the conquest of individualism coincides with the revelation of an illusive ‘happiness’.^1^

Even if the above is true, however Durkheim Theory raises a question. The question here does not refer to causal impact of social group cohesion degree which is considered an established acquisition in the study of suicide aetiology due to various existing empirical supports available. The question, instead, involves the intensity with which modernization and its disruptive effects on social ties influences suicidal behaviour, consequently explaining suicide rates and their evolution in time. In this regard, the theory seems to suggest a constant, proportional, linear increase of suicides as modernization and social fragmentation progresses. *The more* modernization levels and therefore weakening of social ties and social isolation increase, *the more* the individual depends only on himself and recognizes no other rules of conduct than what founded on his private interest*. The more* egoism increases*, the more* social isolation, loss of identity and loss of the sense of life itself increase, and *the more* people commit suicide. Consequently, Durkheimian Theory seems to suggest an interpretation of suicide growth process as susceptible of an progressive, potentially unlimited, increase as modernization increases. Durkheim, of course, never rigorously “formalized” such an idea, but the sense which transpires from his numerous statements seems to leave little doubt. According to Durkheim suicide is a “pathological phenomenon that takes on, day by day, an ever threatening aspect” and it is the Sociologist’s pressing duty to find the means to prevent it (1969 [1897], p. 437).

However, beginning to Halbwachs (1930), more recent studies reveal other different scenarios. In the long run, despite continued modernizing process, several studies, investigating the modernization impact on suicide during a long time frame (from 50 to 100 years and over), found a certain tendency to suicide rates stabilizing (the so called *leveling*-*outeffect)* or even falling in the more industrialized Western world, in particular beginning with the second half of the twentieth century.

How, then, can we interpret these findings? We can hypothesize that a gradual process of adaptation to the stresses of modernization associated to low social integration levels seems to be activated in modern society.

This being stated, first this essays assess the impact of Durkheim’s theory regarding the aetiology and epidemiology of suicide in contemporary society. Secondly, it reviews research founding some evidence for a trend toward suicide rates stabilizing or falling in the western countries more industrialized beginning in the second half of the twentieth century and in some countries even in the first half of the 20th. Assuming a long run perspective, the paper highlights as this tendency may be better understood in the light of the new concept of social systems as *Complex Adaptive Systems.* From this perspective, we hypothesize that the social system, as a *whole*, is able to self-organizing and *adapt* spontaneously to modernization increase by exhibiting *restrained* (non-linear) suicide growth processes, and we root the generating mechanism of this adaptation process in modern society in the non-linearity of social system’s interactions. In the Section titled *Anatomy of Suicide* we expose the theoretical reasons justifying the modeling suicide decision-making process and, therefore, suicide growth process in a nonlinear way, and in particular in a logistic way. Consequently, we use the *Nonlinear Logistic Map* in order to model suicide data in a western modern society such as the modern Italian society from 1875 to 2010. According to May, this nonlinear model, expressing a *restrained* growth process, is *the ruleand not the exception* in the Social Science field **(**May 1976, p. 467). We point out that our analysis is complementary to research that ever since Durkheim has attempted to identify the suitable indicators of modernization for measuring the *degree* of social integration and anomy (i. urbanization rates, divorce rates, unemployment rates, religious commitment) and to correlate these indicators with suicide rate, concluding that domestic/religious individualism has positive effects on suicide. Although we share this concern, our interest is focused on another very basic point. Assuming the Durkheimian perspective according to which modernization/individualization process impacts on suicide curve and the suicide curve constitutes a tangible sign to make inferences about quality of its effects, in the frame of *Nonlinear Dynamical System Modeling* we study *how* the state of suicide population (S_t_) changes in time by formalizing the logic of suicide decision-making process responsible at aggregate level for changes of suicide growth rate by a nonlinear differential equation structured in a logistic way, and in so doing we attempt to capture the mechanism underlying the change process in suicide growth rates (derivatives) and to test the hypothesis that system’s dynamics exhibits on the whole an *restrained* increase process both in suicide growth rates and, consequently, in integrative suicide population as expression of an adaptation process to the liquidity of social ties in modern society. From this perspective, the suicide dynamical analysis becomes an opportunity for a more general reflection on the current configuration of modern society, by relating the Durkheimian Theory with most current visions of modernity such as the Baumanian one.

## Background

### Etiology and epidemiology of suicide in modern contemporary society: Macro Durkheimian Suicidology and its social implications

What is more intimately personal and unique than suicide *act*? The study of suicide as an *act* of individual volition investigates single *subjective* motivations and reconstructs the psychological framework within which extreme suicide decision matures. The motivational study of the suicide *act* attributes to the general category of *loss* (accidents in private life such as loss of a dear one, of a beloved, loss of financial stability, of work, and so on) and to the corresponding feelings of hopelessness, of failure and self inadequacy, the direct underlying causes which explain the individual choice of self-destruction. Yet, how can we answer questions such as the following ones: How and why do suicide rates vary over time (increasing after industrialization process) and space? Why do people commit more suicides in certain social environments than in others? How and why do suicides vary among different social categories? Evidently, the answers to these queries cannot be found in the analysis of single individual suicide motivations, too fragmented to account for suicide rate trends alternating pattern of stability and variability for the same society over time and between different societies. Explaining suicide as collective phenomenon (suicide rates *vs* suicide acts) means therefore to give up an approach oriented to finding simply in the human free will the origin of social phenomena and to recognize the constrictive nature of cultural models in orienting our perceptions and actions, in patterning individual choices and behaviours. So, personal histories and motivations are framed into axiological orientations, that is, into *moral states* of the collectivity whose reference allows us to account for the variability of suicide over time and space, among social contexts and social categories. These acquisitions, that Sociology takes for granted today, are the most significant and most enduring of the Durkheimian Theory. The causes of suicide are identified in structural social forces operating in terms of the logic of *egoism*, *altruism* and *anomie*. Egoism, altruism and anomie are *moral states* of a society, *collective* ways of “feeling, thinking and acting” able to influence the individual and push him to behaviors which are the result of *moral pressures* rather than a mere and free choice of self-determination.^2^

In this regard Durkheim’s acquired data induced him to come to a conclusion which is generally shared by everyone today. In modern society suicide is part of a largest process of social change, being the most tangible signal of modernization process. The weakening of social cohesion secreted by the cultural revolution and by modern individualization processes deriving from the development of industrialization explains the rise in suicides rates in modern societies. Differentiation of functions and interests, pluralism of values, weakening of strong shared traditions and transcendent foundations of the social solidarity reflect on the sense of belonging to social groups and individual identity, hindering strong and stable forms of identification and breaking down up social ties such as the familial and religious bonds which in themselves are for Durkheim able to provide a prophylactic effect on suicide. On the hand, the weaker the ties to groups of belonging are, the less the subject “depends on them, becoming the lone head of himself” and following “only those rules of conduct that are based on his own private interests” (Durkheim 1969 [1897], p. 258). On the other hand, however, the cultural emphasis on personal self-fulfillment even to the detriment of the collective interest generates its own suicidal current in so far as it isolates the individual. In Durkheim’s interpretation, the individual is “freer” but “more alone” and pays for his autonomy of evaluation and action that society indirectly concedes him at a very high price. In this interpretation of modern society we can just find the original inspiration of many actual interpretations of contemporary society. From Fromm to Bauman, the paradox of modern man is the dilemmatic relationship between *freedom* and *security*. First Durkheim introduces us to paradoxes of modernity by a theory of modern society in which, without any long a sense of moral obligation whatsoever towards the groups of belonging, life becomes meaningless, the individual grabs the reins of his existence in such a way that he becomes master of his destiny and such a master of himself that he can terminate his life if he wants. So suicides increase, while, on the contrary, solidarity with groups that one “loves”, protects from suicide attempts by constituting strong bonds of moral obligation, and a worthy end for every efforts of individual activity. The durkheimian idea that the modern process of individualization affects suicide rates by weakening ties to groups of belonging and that “suicide varies inversely to the degree of integration of social groups of which the individual forms a part” or, more specifically, of religious, domestic and political groups, is now an established idea in sociology (Wray et al. 2011). Current sociological analysis has, in fact, empirically supported general formulations of Durkheim’s Theory, concluding that modern domestic and religious individualism (secularization processes, diffusion of a faith lived out of institutionalized dimensions, progressive decreases in marriage and birth rates and increases in divorce rates) has positive effects on suicide (e.g., Agerbo et al. 2011; Breault 1986; Breault and Kposowa 2000; Cutright et al. 2007; Kposowa 2003; Pescosolido 1990; Rendall et al. 2011; Simpson and Conklin 1989; Stack 1983, 1985, 1989, 1990, 1992, 1993, 2000, 2013; Stack and Kposowa 2011).^3^

From this perspective, *EgoisticSuicide,* being characterized by a prevalence of individual interest on collective interest, appears the typical suicide of modern individualistic society.^4^ This concept comes to Durkheim from his analysis of the correlation between suicide rates and the so called *social integrator frameworks*, such as religion and family, the latter treated under the double aspects of marital status and parental status. This analysis suggested to Durkheim the idea that the family and religion are able to exert a prophylactic action as far as they constitutes “a society” and therefore a *value* in itself and for itself: values and collective feelings, shared memories, customs and traditions are its foundations, so that the more intense “the collective life” to which one belongs, the stronger is the bond that unites the individual to his domestic and religious community and the preventing effect on suicide. This explained why in modern society a greater religious and domestic individualism determined a suicide increase (Protestants vs Catholics, singles and unmarried vs married, married without children vs married with children, divorcees vs married…).^5^

As was mentioned above, Durkheim’s acquisitions have been confirmed in various studies throughout time. In this regard, from an epidemiological point of view, the same regularities observed by Durkheim over one hundred years ago still exist today. Statistics today present greatest suicide rates for the same religious groups and marital status that were treated by Durkheim in his sociology study: protestants, singles, childless married couples, widowers, separated and divorced people compared to married couples, divorced males. On the one hand, research has showed that Catholic countries have lower suicide rates than Protestant countries (i.e. Pescosolido and Georgianna 1989; Hood-Williams 1996), marriage is a preserving factor regardless of age and socio-economic status and suicide trends decrease within fertile families (Lorent et al. 2005, in a comparative european study; Rendall et al. 2011). Children play a protective role for the male and the female as well. In fact, married women without offspring have a higher suicide rate than married women with offspring. Therefore, as Durkheim believed, it is the *familysociety* and not the *conjugal society* that has a protective role against suicide, and this capacity is greater the more numerous and united the family actually is.^6^

On the other hand, there is yet another acknowledgement in favor of Durkheim’s theory which is being frequently confirmed today. Much of the discussion of social integration and suicide uses divorce rates as a key indicator of degree of social integration. Even after Stack’s last systematic review (Stack 2000), research has continued to document a strong association between divorce/separation and suicide (Wyder et al. 2009). Investigations based on individual level data showed that divorced people tend to have a higher risk of suicide than married people. For example, divorced Americans tend to have a suicide risk double that of their married counterparts (e.g., Kposowa 2003; Stack and Scourfielf 2013). Investigations based on aggregate-level data found a robust relationship between divorce rates and suicide rates. Confirming the results of preceding investigations (for reviews see Stack 2000), a very recent study conducted on suicide rates in Denmark from 1906 to 2006 offered the strongest support to date in support of a social integration model based on long *time seriesdata* on suicide and divorce (Agerbo et al. 2011). It found, in fact, that marriages decreased suicide (men seemed to benefit more from marriage than women: a 1 % increase in marriages reduced suicide by 0.77 % for men and by 0.63 % for women) and the trend in divorce, in particular, offered accurate predictions of suicide (total, male and female) throughout the century. In addition to Durkheimian *Egoistic suicide* conceptualization, data, today as in the past, seem therefore to recall the conceptualization of *Anomic suicide* and the idea that the different protection that marriage itself ensures to the two genders would then depend on their correspondingly diverse *moral constitution*.

A*nomic Suicide,* also typical of modern societies, stems from a loss of society’s moral regulation power. Here, interpretation is influenced by Durkheim’s convictions regarding human nature, a nature capable of unlimited passions which only strict obligatory social rules are able to control, safeguarding life in society. As we know, it refers to the structuring of the collective state on the basis of dominant principles that encourage the individual to transcend and challenge culturally ends and means. Clearly, this does not mean that “ends” and “means” are left to the moral autonomy of the individual rather than to the community, but that they are simply of no regulatory significance. This peculiar axiological configuration produces suicide effects insofar as the weakening of the power of rules, creates a discrepancy between the individual’s aspirations and their satisfaction.^7^ According to Durkheim, this would explain the increase in suicide rates produced not only by economic downturns but also by “crisis of prosperity” that alter the collective order.^8^ In this sense, anomic suicide is the most typical suicide of our times, marked by rapid, unregulated and unchecked economic shifts. From this Durkheimian perspective, also conjugal anomie is substantially explained in the same terms as economic anomie because of deregulation between aspirations and satisfactions produced from divorce in the human passionate life. In particular, as far as the two genders are concerned, divorced men are more likely to self-destruct than divorced women because they are more subject to the *mentalism* of sexual love and therefore more needful of passion regulation.^9^

As regards economic anomie, economic indicators such as unemployment rates, pro-capita income and gross national product were widely used to test Durkheim’s hypothesis. Some studies found an inverse relationship between suicide rates and economic growth rates (Gross domestic product (GDP per capite) and a positive relationship with unemployment rates (Blakely et al. 2003; Granados 2005; Ying and Chang 2009; Luo et al. 2011; Blasco-Fontesilla 2012; Reeves et al. 2012; De Vogli et al. 2012, 2013). However, divorce, used—we repeat—as indicator both of integration degree of domestic society and of conjugal anomie, and religious affiliation have been found to be the strongest determinants of suicide rates, even while controlling the incidence of a great many economical and modernization factors, such as unemployment rates, income levels, urbanization rates, female worker quotients and population growth rates. According to researchers, this confirms the protective effect of domestic and religious integration (Islamic religion as well, Lester 2006; Stack and Kposowa 2011).

Durkheimian Theory has not been only supported in its direct original formulation. Interestingly, current sociological research has also supported one of the main theories of strong Durkheimian inspiration, the Gibbs and Martin’s *Status Integration Theory* (1964). Here suicide is correlated to role conflict, to poor status integration and to stress associated with having to face mutually conflicting behavior expectations ending up by compromising stable and long lasting social relationships. Current sociological research on conflicting and statistically infrequent status/role sets (i.e. being a female in the labor force or wife-mother in the labor force) largely confirmed the positive impact of low status integration degree on suicide (i.e. Cutright et al. 2007; Fernquist 2009).

In conclusion, after more than a century, we can still be agree with Breault and Barkey (1982) in stating that Durkheim’s study on *causes* of suicide as collective phenomenon stands very well over the years. Insofar as a lack of social integration entails at the same time a lack of social regulation as well, *Egoisticsuicide* appears—we repeat—the typical suicide of modern society. So, sociological analysis of suicide becomes an opportunity for a more general discussion on the relationship between *individual* and *community,* individual identity and collective identity, human nature and social normativity. These are relationships addressed by Durkheim, becoming a touchstone for contemporary sociological studies on social integration and social implications of moral individualism. Durkheim masterfully captures the perverse aspects of modern cultural emphasis on individualism, on personal self-fulfillment even to the detriment of collective interests, and attempts to persuade us that social groups cohesion and a strong sense of social belonging are able to offer to each individual an indispensable human environment more than it denies and limits his freedom. For him there is no doubt that strong ties between the individual and society strengthenes the reasons for living, whereas their loss is equivalent to losing the sense of life and identity. Today, a well as for Durkheim, the empirical findings of current research on suicide lead us to the dilemmatic structure of relationship between individual and community. As sociologists, looking at suicide as collective phenomenon means, in fact, looking at the *darkest side of freedom*, at the unintended consequence of a structuring of society which in itself and for itself possesses however an undeniable ethical value.

### Suicide: an emergentist versus a linear approach to social change processes

As was stated above, causal impact of social group cohesion degree on suicide is an established acquisition in the study of suicide aetiology, due to various existing empirical supports available.

However, if it is true that industrial development inflates suicide rates by facilitating social disintegration, nevertheless, the modernization process has been shown to produce with *utmost intensity* certain pernicious consequences, weakening traditional life systems and sacrificing always more victims on the altar of modernity, especially in the first phase of its development. Beginning to Halbwachs, *despite continued modernizing process* (i.e. increase of urbanization and divorce rates), several studies investigating the modernization impact on suicide during a long time frame (from 50 to 100 years and over) actually found some evidence for a trend toward a suicide rate stabilizing (*leveling*-*outeffect*) or even falling in the western countries more industrialized beginning in the second half of the 20th century and in some countries even in the first half of the 20th. In other words, suicide growth rates seemed not to increase in a constant, linear, proportional way to modernization and social fragmentation process increases.^10^

Often ignored by current literature, Halbwachs’ Theory (1930) is highly relevant today in interpreting suicides in the our post-modern era. Transcendent in relation to single individual volitions, suicide with Halbwachs remains arguably the distinctive product of modernity. Therefore, his most original contribution to the interpretation of suicide consists in having theorized first an adaptation and suicide rate stabilization process in response to modernization progress in the long run. That is, he sees the growth in sucides as a not unlimited process. In fact, working over a quarter of a century after Durkheim, he found that suicide rates, which had increased in the latter half of XIX° century, tended to stabilize and even decrease in some more idustrialized countries (including England, Belgium, Norway) in the early twentieth century, whereas they tended to increase in countries in initial industrial development, involved in progressive depopulation and weakening of traditions. This process led Halbwachs to assume that, as high levels of economic and social development were reached, each nation would lend itself to a maximum suicide rate (whose variability was cultural and social) which once attained would not be exceeded (1930, pp. 100–104). His *Law of Convergence* among suicide rates in more industrialized nations (tending to stabilization) and suicide rates in developing nations (tending to increasing) allowed Halbwachs a broader commentary on the effects of industrialization in what we would call the “long term”.^11^ In the long term the initial shocks of modernization would gradually overcome, and social actors would *adapt* to the stress deriving from industrial urban society (1930: 484–490). The benefits of industrial-machine production would offset social isolation effects induced by low levels of domestic and religious integration.

Similarly, Krujits (1977) found that the figures for suicide in the centrally located countries of Western Europe and in many countries within Anglo-Saxon culture sphere (i.e. United States, Canada, Australia and New Zealand) showed a stabilization or decline in the suicide rate after the turn of the nineteenth century where industrialization process was already at its culminating point. According to the author, this was “an indication that industrialized Western World was growing towards a new equilibrium in the first half of the twentieth century” (1977, pp. 55–56).

Thomas and Gunnell (2010) confirmed Kruijt’s finding by analyzing age standardized suicide rates (for age ≥15 years) in England and Wales. They steadily increased from 1861 to reach a peak of about 36.0 in 1905. Rates then decreased in 1917 (during World War I), increasing to reach a second peak in 1934, coinciding with the Great Depression. Subsequently they declined (although these declines were interrupted by small increases in the 1950s and 1980s). The lowest recorded rates were in the 21st century: the lowest male suicide rate (11.6 per 100,000) and the lowest female rate (3.2 per 100,000) was seen in 2007.

In his study on suicide rates in Finland from 1800 to 1984 Stack (1993) found that a 1 % increase in urbanization was associated to a 0.22 % increase in suicide rates when considering nineteenth century rates only and to a 0.12 % increase when considering data from the first half of the 20th. The *slope* of the modernization and social fragmentation thusly decreased. According to the author, therefore, although a positive impact of modernization on suicide was still observed (the slope was not zero), at the same time there was “some evidence for a trend toward a leveling-out effect” (Stack 1993, p. 145). By using a log-linear Poisson regression model on suicide rates in Denmark from 1906 to 2006, Agerbo et al. (2011) found the parameter associated with the time-trend was negative for both genders (φt = −0.14), which “primarily reflected the declining number of suicides in the later part of the period” (p. 634). Furthermore, the analyses suggested that the impact of divorce on suicide, although found, was declining.

By analyzing suicide rates in 105 countries of the World from 1950 to 2009, Värnik (2012) found generally the suicide trend was downward in Europe and there was no Western European more industrialized states in the world top ten for suicide rate. Suicide mortality has shifted from Western Europe to Eastern Europe and to developing countries of Asia (China and India). Similarly, several studies, by analyzing suicide rates in more industrialized, new and early members of the EU, found overall suicides were stabilizing or falling before the economic crisis in 2008 (i.e. Innamorati et al. 2010).

For our part, by using the modern *Bayesian Change*-*Point Analysis* on Italian suicides rates from 1864 to 2005, we found this general trend in Italy before 2008 (Condorelli 2013a). The analysis suggested a *Model with 5 change*-*points:* mode at *r*_1_, *r*_2_, *r*_3_, *r*_4_, *r*_5_ = 13–31–98–121–133 corresponding to 1876–1893–1961–1984–1996. These results showed a very complex scenario. The first change-point (and therefore the ‘*first wave*’ of suicides) was found just after the *feverish triennium*, that is the period from 1871 to 1873 in which great industrialization in Italy originates (De Rosa 1980). From the Durkheimian perspective, therefore, this transformation explained the wave of suicides after 1876, 1889, etc. Furthermore, always in accordance with the Durkheim’s theory, suicides reached the lowest values during the World War II and soon afterwards began increasing again until 1961 with the contemporary rise of the industrial production index. However, if until 1961 suicides rates increased as industrial development increased, after 1961 and the *economic boom,* they declined, and when they *began increasing again*, after 1984, they did not reach the maximum levels attained formerly, before World War II (suicides steadily increased from 1876 to reach a peak of about 10.5 in 1927 and 10.03 in 1930; rates then decreased and particularly from 1961 to 1984 suicides exhibited a maximum rate of about 5; subsequently from 1984 to 1996 they increased coinciding with Italian monetary and financial crisis in the 1980s and 1990s, and however the maximum peak was of about 7,2 in 1993; from 1996 to 2010 suicides exhibited once again a maximum rate of about 5). From our perspective, the observable change of suicide trend since 1961 showed a dissonance with Durkheim’s theoretical prediction. Increases in economic prosperity and consumption styles seemed to be a deterring factor on suicides. Interestingly, although in Italy from 1995 to 2010 overall suicide mortality rates per 100,000 inhabitants appears on the whole to be decreased (the data, presently available until 2010, allows us to draw only preliminary indications on suicide trend after economic crisis in 2007: from 2005 to 2010 suicide rate seems to remain constant with about 5 suicides on 100,000 inhabitants, *ISTAT* 2012), there is a trend significantly different if only suicides due to economic reasons are considered. Upon the onset of the financial crisis in 2007, De Vogli et al. (2012, 2013) found suicides due to non economic reasons remained stable, while suicides due to economic reasons increased^12^. Compared to downward trends in the pre-crisis years, rises in suicides was found in European economies as Greece and Spain after crisis economic from 2007 to 2010 (De Vogli et al. 2013).

In summary, these long-run findings impose an interpretation. From this point of view, we believe that they seem to credit what Halbwachs maintained. In other words, they seem to legitimate the hypothesis of a *restrained* suicide growth process and therefore to cast in doubt the possibility to find an explanation within the classical conception of social change which assumes all systems, and social system too, as systems being characterized by interactions based on linear proportionality between cause and effect. Instead, from our perspective they may be better understood in the light of the new concept of *complex adaptive systems*, systems which are composed of several elements interacting in a nonlinear way and, consequentially, subjected to a nonlinear, *emergentist* process of social change. This new approach had many implications for Social Sciences.

### Society as Complex Adaptive System or far from equilibrium system the rejection of linearism and reductionism of Newtonian–Laplacian epistemological paradigm

The concept of social system as *complex system* is relatively new in Sociology, but it has been from its outset sufficient to reconsidered some aspects of Parsons’ functionalism to which the success of system concept in Sociology is nevertheless due. From this new theoretical perspective, the critical point has been identified in the equilibrium concept considered from Parsons the foundational property of social system such as *ordered,stabilized* or *in equilibrium* interweaving of interactions embedded in social structures. Equilibrium as order system state or system stability (steady state), emphasizing the tendency to self-maintaining and returning to a particular state if disturbed, showed in fact to be still influenced by *epistemological deterministiclinear Newtonian*–*Laplacian paradigm* of classical science, a paradigm that the *New General System Theory* (*Complexity and Chaos Theory*) has today questioned encouraging its critical review in all sciences including Sociology. The more Classical science looked at systems as governed by a linear causality, by proportional relationships of cause and effect, and maintained in stable order by control mechanisms such as negative feedback, liable to ensure prediction and control over events, the more contemporary scientific reflection, matured in the field of Natural Sciences (Physics and Biology), has gradually revealed the limits of the mechanistic and reductionist paradigm imposed by Newtonian Physics. Consequently, the macro-sociological analysis of the social system has proceeded to revise inside the linearity option involved in the structure of social interaction processes, and especially to cancel the claim constituted by the equilibrium concept (Bailey 1984). On the one hand, the revision was needed because the equilibrium concept seemed misleading as it was used by Parsonsian functionalism, alluding inappropriately to a state of order or stability of the system rather than to a state of maximum entropy, maximum disorder or system death according to its more correct scientific meaning established by Thermodynamics. On the other hand, even starting from the consideration that Parsons, as Bailey pointed out (1984), uses the concept of stable or in equilibrium system in the meaning of *homeostatic* and not static *system*, the revision was needed because this conception is associated to the idea of a ordered change process, “following a determinate pattern rather than random variability relative to the starting point (moving equilibrium, which is exemplified by growth)” (Parsons and Shils 1951, p. 107), endorsing linear social interaction and change processes. Because of its implications, in neither of the two senses (stability/homeostasis or maximum entropy) equilibrium did it appear however appropriate in describing social systems as far as they are *open systems*.

After von Bertalanffy (1969), Prigogine and Nicolis (1977), Prigogine and Stengers (1979, 1984), Maturana and Varela (1984) the qualification of real systems as open systems, which exchange information and energy with external environment, has in fact fixed the foundational system properties in an *instability condition* rather than in the tendency to asymptotic stability or in the tendency to the state of maximum entropy, of maximum disorder with minimal internal differentiation/organization (equilibrium in a thermodynamic sense, which is appropriate in describing closed system but not open systems such as social systems are and we ourselves are, from a biological standpoint and in our cognitive processes as well). This acknowledgement, which in Sociology meant going beyond Parsons’ functionalism (Bailey 1984) without renouncing to a macrosociological analysis of society as a whole, is the central acquisition of the current scientific-epistemological approach to the study of systems as complex systems.

As was said above, the notion of complex systems is relatively new in the Social Sciences, but not in the Natural Sciences. *Complexity epistemological paradigm* reflects on the structure of the relationship among elements constituting a system. The novelty lies in a dual acknowledgement: the properties of *non linearity* of interactions among system components (non proportional relationship between cause and effect whereby “small” initial variations in cause may produce “big”, unexpected effects), and the properties of *self*-*organization*, *adaptive evolution* and especially *unpredictability* of systems in their self-organizing process due to interactional nonlinearity and positive feedback. In brief, looking at systems as complex systems means that they are open systems, made up of many interacting elements in a non-linear way, and *far from equilibrium* systems (in a thermodynamic sense, namely *maximum entropy*) or *dissipative structures*, that is, instable structures, *at the edge of chaos* (Kauffman 1995; Langton 1990; Waldrop 1992), in an intermediate state between complete order and complete disorder, able, in this intermediate state, to self-organize and evolve for adaptation in response to environmental perturbations, producing *emergence,* unexpected and unpredictable changes as result of nonlinearity of interactions and positive feedback. So, s*elf*-*organization* refers to the spontaneous emergence of order in complex systems, an order of non-equilibrium but also a non-static, unstable and unpredictable order, different from the state of asymptotic stability assumed from classical science. In a system governed by a linear causality and negative feedback the whole dynamic of evolution tends to go off in a stable order and there is no place for *surprise*, for unexpected and surprising changes of internal system structures. Instead, in an anti-reductionist perspective, nonlinear interaction among system’s constituent parts creates *spontaneously* self-organization, new patterns of relationship, a continuously new order, an *emergent* effect being unexpected, surprising, unpredictable as its properties are properties of the “whole” and not reducible to the *sum* of individual component behaviours or rather to the sum of individual interactions among components, considered one by one.^13^

This paradigm, today, enjoys wide diffusion in the Social Sciences as well, due to its ability to describe traits which appear peculiar to social systems as well as physical ones (self-organization, emergence, evolution for adaptation, irregularity and change unpredictability) (Ball 2012), unable, in this case as well, to be comprehended by traditional approaches based on the deterministic linear Newtonian-Laplacian paradigm (Condorelli 2013b). As we said, the macro-sociological analysis of social systems has today no problem in going beyond Parsons’ functionalism and recognizing social systems’ assignment of *dissipative structures* or *adaptive* (Miller and Page 2007) and *autopoietic* complex systems, identifying their properties in being, as open systems, far from equilibrium systems, intermediate between order and disorder (neither too regular and predictable such as crystal molecules nor too random and chaotic such as the molecules of a gas tending toward entropy). They are unstable systems too, but able to adapt to stresses coming from environment by generating spontaneously (from inner guidelines rather than the imposition of form from the outside) self-organization and evolving to a new interaction structure, to a new pattern of social expectations, in a relentless and unpredictable production process of new structures, new communication through communication (Luhmann 1984, 1986).

In the current approach to social system nothing remains of the mechanistic and reductionist epistemological paradigm engendered by Newtonian physics, with its linear determinism (able to ensure instances of predictability and control over events). The new approach to Society as a complex system rejects reductionism and mechanicism, addressing the classic Sociology questions of micro–macro relations (the relationship between system and its parts) from the perspective of *systemic connectionism*. From this perspective, the interactive relationship does not simply unite the parts like in an aggregate but mixes them up in a super ordered whole. In other words, they become a *system* in which and through which components are connected to each other and are considered a totality rather than separate entities. The rapport between the parts and the whole, at this point, implies a new determination of causal relationships. The *whole* influences the parts/components of the system, and every element can act upon the whole and can modify it (*bottom*-*up process*), pushing it into a new order, which will be maintained until a new disturbance pushes it to a new and unpredictable evolutionary direction, in a new pattern of social expectations which in turn connects the parts in a new form (*up*-*down process*). On one hand, therefore, the self-organization process is a *deterministicbottom*-*up process*, on the other hand local interactions, extending to the whole system, generate, as result of nonlinear social interactions and positive feedback, *emergent* patterns, unpredictable and unexpected global effects which are beyond the intentions of each agent and which can not be explained reducing them at the properties of individual interactions since they constitute an “effect of the system” as a whole, as an organized and dynamic collective entity. In short, this perspective leads to re-specify the classic concept of *inherent indeterminacy* of human behavior. Complexity approach acknowledges this inherent indeterminacy. However, here this concept is far from meaning that any order or any structural explanation of social life can not be found and that a dice toss is the fundamental engine driving social processes. According to Huckfeldt, for example, this is a *epistemological naivety* associated with an earlier era (1990, p. 431). “It is mistake”, Huckfeldt noted, “to argue that seemingly infinite complexity is necessarily a repudiation either of deterministic argumentation or of a structural interpretation of social and political life” (Huckfeldt 1990, p. 429). Rather, from complexity perspective, this concept means acknowledging that complex and even seemingly stochastic behaviour can be fully generated by a determinate structure underlying the logic of human behaviour and, therefore, its indeterminacy is just inherent to a *particular structural mechanism* underlying social interaction processes (*cit.*, p. 429), whose logic revealed now a nonlinear structure. These new idea was synthesized in the *deterministicchaos* concept. As a result, the goal of social sciences was re-specified as well. From this point of view, Social Sciences have to identify the deterministic structure and logic underlying human behaviour “including the logic and structure of indeterminacy” (*cit*., p. 431), which therefore should not be longer an metaphysical element but a valuable conceptual tool in the analysis of social life. In other words, today Complexity epistemological paradigm emphasizes the awareness that, although we can not *predict* social phenomena, we must to attempt to *understand* underlying mechanisms governing social phenomena by modeling nonlinear social interactions (see also Bak and Chen 1991).

In conclusion, to apply the concept of dissipative structure or *complex adaptive system* to the study of society means looking at social systems as “inherently historical entities” whose evolution “is driven as much by internal instability as by external perturbation”(Harvey and Reed 1997, p. 306), using environmental feedback for learning and adaptation. And the same conditions of nonlinear interactions or sensitive dependence on initial conditions observed for natural systems is the foundation for their historicity. This realization introduces us to an *emergentist* conception of social change which celebrates *discontinuity* and *unpredictability* and *uncertainty* of the process (Prigogine 1997) in as much as it is governed by non-linearity underpinning the deterministic mechanism of evolution. Compared to linearism, the directional shift is, therefore, substantial. The more linearism describes social systems implying a process of change where constant proportionality relations between cause and effect (linearity logic, the more… the more, the more… the less) turn out in the conceptualization of a regular and predictable process with linear trend patterns (constant growth/decline parameters) excluding the possibility of irregularities or temporal discontinuity, the more social sciences had to disavow the pervading existence of these social change processes. It was this conceptual model with its consequential use of linear equations which led Malthus to predict the exponential population growth concluding that it would be unsustainable when compared to the arithmetic growth of resources. On the contrary, today, several studies show the validity of the new conceptual model. They present, rather, the effectiveness of nonlinear models in formalizing and describing discontinuous processes of social change beginning with the population’s evolution itself and market instability, to go on to phenomena such as political revolutions, voting and electoral shifts, crime dynamics, urban growth, spread of innovations, adolescent childbearing, marital instability, authoritarian attitudes (on these issues, see: Saperstein 1984; Tsebelis and Sprague 1989, 2010; Brown 1991; Huckfeldt 1989; Priesmeyer 1995; Condorelli 2013c; Dendrinos 1992; Dooley et al. 1997; Gottman et al. 2002; Guastello and Guastello 2008). Many of these studies found, in particular, that social systems, with reference to their movements over time, fluctuate between different critical points (*bifurcation points*) rather than follow a direct path, presenting a bounded development process. In this process, human interdependences are structured according to a non linear logic of the *logistics* type where the interplay among factors that promote growth and factors that act as restraints (such as in a game competition) contrasts the idea of a regular linear or exponential trend, which is the expression of cause and effect constant proportionality logic, and is able to result in unpredictable outcomes of social interaction relationships and irregular and instable trends of social change process (even chaotic processes).

In closing, although some criticisms were advanced [for example, some researchers doubted that science can achieve an unified theory of complex systems able to go beyond some general principles, as complexity researchers such as Bak, Holland and Kauffman suggested, considering that it implies a *reductio ad absurdum* (Anderson 1972); and some found themselves uncomfortable with the romantic Prigoginian idea that the vision of a complex, unpredictable, without certainty world but able to emphasizes the *re*-*enchantment of nature* is more comforting than the scientific vision of a predictable, timeless, deterministic world; for a review see Horgan 1996)], nevertheless *Complexity* point of view seems to lead to a more realistic awareness of working and evolution mechanisms of the Natural and Social Systems compared to traditional science. By detecting the rule in *discontinuity,surprise* and *uncertainty,* it allowed us to bring out of the limbo of the brain teaser (Gleick 1987) observed social discontinuity (market and international political competitions instability, electoral volatility, social control processes, spread of social epidemic), just like Natural Sciences have brought out of the limbo of the brain teaser observed natural discontinuity such as atmospheric and fluid turbulence. A last thought goes, therefore, to a potential unification of the Sciences implicit in the complexity approach. What has been traditionally considered separate objects of study—on one hand, *free human acts*, with their uncertainty and unpredictability, and on the other hand, *nature*, with its inner order—has created a gap between the Social and the Natural Sciences. The *Complexity Theory* (or *Nonlinear Dynamical Systems Theory*) shows this *gap* to be largely artificial, redeeming the Social Sciences from being a *minority* science, in Kant’s terminology, or in Kiel and Elliott’s modern terminology, a “*scientific stepchild*” compared to the so-called “*hard*” sciences (Elliott and Kiel 1997, p. 3).

### Social complexity and suicide: the research hypothesis and its theoretical justification

As we said, we believe that the empirical long-run findings above mentioned can be better understood in the light of the new concept of social systems as *complex adaptive systems*. From the perspective of suicide, social systems seem to confirm essential traits of complex systems. Suicide trends seem to lead us to think that the criterion leading to actions in an interaction system based on weak ties is not necessarily characterized by the *proportional* increase of identity loss and meaninglessness of existence as modernization and social isolation condition increases, and that, instead, individualism and *liquidity* of social ties characterizing our contemporary or post-modern society (Bauman 2000) has “strengthened” up to the point of neutralising, to a certain degree, that disintegrating valence regarding identity and sense of life which, according to Durkheim, is the first propeller toward self destruction. As well as Halbwachs, we can be led to hypothesize that after the initial shocks of modernization, a gradual process of *adaptation* to the stress of modernization associated to low social integration levels is activated in contemporary modern society. That is, many people get used to living with the progress, with the perverse consequences of organic solidarity which become gradually liable to be assimilated and absorbed as parts of a ‘normal’ everyday life. Durkheim said: “our sensitivity” is a bottomless abyss which nothing can fill. However, if Durkheim modern man lives suffering the tragedy of his freedom, here the hypothesis is that in our post-modern society, being characterized by a increasingly fragmentary and uncertain sociality (frailty of human bonds continues increasingly to undermine all social institutions since their own constitution, beginning with the family and the more intimate matrimonial or couple relationships, as to be itself become an institution; Bauman 2000), this sensitivity seems to have increased to such a point that it can eventually enable a sort of *immunization* against the weakening of social ties and the emergence of a new pattern of social expectations which restrains the impact of the factors that lead to suicide and promote its growth. In other words, individualism does not destroy identity and the sense of life with the intensity which Durkheim had originally expected because, by applying conceptual categories of dissipative structures or complex adaptive systems, the social system as a whole seem to able to self-organizing and adapt spontaneously to modernization increase. Likely, adaptation to weakening of social ties processes in more industrialized western countries may be encouraged from benefits of industrial and economic progress. They may to offset modernization stress: improvements in living conditions, changes in istitutions as welfare and health services (social services for the aged, working mothers…) may help to accommodate the modern person and, in so doing, create a less suicidogenic environment. However, we agree with Krujits (1977) in thinking that changes in welfare and prosperity can not be the sole explanation for adaptation. One essential condition is the emergence of a materialistic culture, an explicit change in mentality, geared more towards consuming than towards family and working, traditional values and standards. Economic prosperity can be able to encourage this mentality, so that the fragility of social bonds may no longer be lived in a desperate form. From this perspective, as we said, at the bottom of the explanation there is still that same *human sensitivity* leading Durkheim to say that we are a bottomless “pit” that nothing can fill and ending to make normal social fragmentation too. So, new cultural models, new models of social expectations may emerge, and people may *adapt* and become less inclined to suicide. In other words, we are saying nothing but suicide growth may be characterized by a *sensitive dependence on initial condition*. For this same reason, if a materialistic mentality may be able to limit the suicide growth, a suicide increase may be expected when materialistic need are not satisfied, namely in crisis economic conditions (as suicide increases after economic crisis in 2007 show).

To sum up, in the framework of complex social systems approach where *uncertainty* is the “rule of the game” of social interactions process dynamics, we hypothesize that immunization and adaptation to the individualization process as emergence of a new pattern of social expectations, absorbing in ‘normality’ the liquidity of social ties, and consequently a nonlinear, non constant and non-proportional suicide growth rate, may to represent the *spontaneous self*-*organization* of social systems, the *unpredictable*, *surprising*, *emergent effect* produced from the system as *a whole* by effect of nonlinear interactions among its components/agents.

On the one hand, this perspective lead us to revaluate Halbwachs’ Theory (1930). From our point of view, just for this insight of adaptation process Halbwachs could be considered a forerunner of the dissipative structure concept, the same way as Prigogine considered Durkheim, interpreting particularly the labor division process as a prove of spontaneous self-organization process of social system in response to society’s moral and material density increase.

On the other hand, although Halbwachs had guessed there was an adaptation to individualism effects, the mechanism of social interaction which justifies this process remained still undetermined. In this regard, we think that the current complex systems paradigm can help us to take a step ahead. The step ahead is the fact that today we can be able to better understand the underlying generating mechanisms of this process insofar we can root it in the conception of a *new* General Theory of Systems such as dissipative structures and, therefore, in the *non*-*linearity* of social system’s interactions.

In order to support this interpretation we propose to modeling the logic of suicide decision making process responsible for longitudinal change in suicide growth rates by a differential nonlinear equation able to model *restrained* population growth processes, that is, by a nonlinear equation which is structured in a *logistic* way. Consequently, we attempt to apply the Logistic map to an empirical suicide growth process in modern society, namely to suicide trend in modern Italian society from 1875 to 2010.

### Dynamical System Analysis and nonlinear *Logistic Model*

*Dynamical System Analysis* is interested in *how* the system’s state changes in time. From a sociological perspective, the dynamics of a social phenomenon at aggregate level (i.e. marriage, divorce, suicide, politics voting…) expresses the result of individual decision making processes and therefore of social interaction processes. Collectively they produce an aggregate configuration of social phenomena. Insofar these decision-making processes can be affected by broad social and cultural factors (as well as in the passage from pre-modern to modern society), dynamics of social phenomena at aggregate level expresses in a tangible way the onset of possible changes in the structure of social interactions and allows us to make inferences about cultural changes which can have influenced these possible changes in individual and social decision-making processes. Therefore, making a dynamical analysis of social systems expresses the attempt to model the structure and the logic of human behavior and underlying mechanisms governing social interactions, which are responsible for changes in social phenomena at aggregate level in time.

This being stated, the simplest process of change at the level of a natural or social phenomenon is constant growth or decay. Constant growth indicates that some population, say an ecological population or a social group—i.e. political party, deviant group, suicide group, consumers, married and divorced people, etc.…—increases its membership at a constant over time rate. In such a case a certain number of new elements adds to the group each time period. Constant decay expresses the reverse concept, that is, the group loses the same number of elements each time period.

To represent change in the membership of some group (in our case, suicide group) the term *dy/dt* is used to refer the rate of change or growth rate for that population group. As an example, y is the level of some population group Y in time period t. The term *dy/dt* is a derivative and it is a function that describes longitudinal change in the levels of Y within the population. If Y neither gains new members nor loses old members, then the derivative is equal zero. If, on the other hand, it gains (or loses) a set number of members each time period (a net gain or net loss), then the rate of change would be constant. A constant rate of change is described mathematically as

1 $${\text{dy}}/{\text{dt}} = a$$

or in discrete terms

2 $${\text{y}}_{{{\text{t}} + 1}} - {\text{y}}_{\text{t}} = a\quad (\varDelta t = 1)$$

where *a* is a constant and a parameter of the model. The graph of the function (placing x_t_ values in abscissa and the derivates of function in ordinate) is a flat line. For this simple model, the over time behaviour of equation or the sequence (*trajectory)* of solutions generated by the constant growth or decay model forms an up or down straight line (as a plot of the integrated population versus time t shows). As we know, solutions of a differential or difference equation can be approximated by Euler’s method and they are much more accurate as smaller *h* integration interval is. In some cases, exact solutions can be obtained using algebra and obtaining mathematical general law. In this linear case, exact solution is the following general law:

3 $${\text{y}}({\text{t}}) = {\text{y}}_{0} + a{\text{t}}$$

or, using the discrete notation,

4 $${\text{y}}_{{{\text{t}} + 1}} = {\text{y}}_{\text{t}} + a$$

There is no other possible variations in the structure of this type of dynamic as long as parameter *a* is constant (Brown 1991). However, the substantive application of constant gain or loss as a model may be quite limiting with regard to most natural and social processes. A more interesting model is the Malthus model including a description of the growth rate as dependent of the number of people in the population in each previous time period t. This model is herein interesting for us, because Logistics is just the result of an opportune adjustment by Verhulst of the Malthus’ law for population growth. It is a simple differential equation able to model population changes from t time to t + 1 time by a mechanism expressing a ‘free’, unlimited, growth process (May 1976; Braun 1993; Kostelich and Armbruster 1996). Indeed in the Malthusian Growth Model the growth population mechanism equals *a*y_t_, and the growth rate *a* is constant, that is, it does not change with either time or population. Therefore the following differential equation governs the population growth mechanism:

5 $${\text{dy}}/{\text{dt}} = a{\text{y}}_{\text{t}} ,\quad a = {\text{constant}}$$

In discrete terms, adapting the difference equation notation, we have the following equation:

6 $$\varDelta y_{\text{t}} \left( {{\text{or}}\,{\text{y}}_{{{\text{t}} + 1}} - {\text{y}}_{\text{t}} } \right) = a{\text{y}}_{\text{t}} \quad (\varDelta {\text{t}} = 1)$$

where Δy_t_ is the change in y population between two adjacent time period (y_t+1_ − y_t_) and y_t_ is the population at the beginning of the *i*-th interval of length 1 (Δt = 1). Population at time t + 1 depends solely on population at time t. It is linear function of y_t_ because it is proportional to y_t_ by a constant fraction or relationship of proportionality (*a*). The graph of the derivative function is an upward or downward straight line. Consequently, any population satisfying the Malthus’s population growth law grows *exponentially* with time (trajectory of solutions is an up or down curve line). Indeed, its exact solution is the following equation:

7 $${\text{y}}({\text{t}}) = {\text{y}}_{0} e^{\text{at}}$$

where y_t_ is the variable indicating the value of population at time t, y_0_ is the initial value of population, and *a* is the constant growth rate of population. The exponentials equation “represent the solution of a linear one-dimensional differential equation and as such arise in a variety of circumstances in which the rate of change of a variable is proportional to the value of the variable” (Kaplan and Glass 1995, p. 157). As it is known, the exact solution of Malthusian model can be written as

8 $${\text{y}}({\text{t}}) = {\text{y}}_{0} {\text{b}}^{\text{t}}$$

where *b* is the Anti-logarithm of *e*^*a*^ (if *a* > 1, *b* = 1 + growth rate *a*)

Adapting the discrete notation, the Eq. (7) is equivalent to

9 $${\text{y}}_{{{\text{t}} + 1}} = {\text{y}}_{\text{t}} + a{\text{y}}_{\text{t}}$$

or again

$${\text{y}}_{{{\text{t}} + 1}} = b{\text{y}}_{\text{t}} \quad {\text{where}}\,b\,{\text{is}}\,{\text{y}}_{{{\text{t}} + 1}} /{\text{y}}_{\text{t}}$$

if *a* > 1, *b* is 1 + *a* (1 + growth rate *a*), if *a* < 1, *b* is inferior to 1 ^l^. In turn, the equation y_t+1_ = *b*y_t_ is equal to

$$y_{t + 1} = y_{0} b^{t} \quad \left( {b = 1 + a} \right)^{n} .$$

As we said, the Malthus model structures an unrestrained growth process. However, when the population gets too large, Malthus model it can not be very accurate, since the environment cannot support unlimited growth due to limited environmental resources. Several factors discourage a further growth (limited living space and resources, competition among individual members for limited resources). The Verhulst’s correction to Malthus model avoids this problem, since it reflects the fact that the population growth is the result of opposing forces: the forces ecouraging growth and the forces acting as a restraint. Therefore, it includes a restraint preventing an unlimited growth mechanism. This is obtained by adding to second part of Eq. (5) and its discrete version (6) a negative term, the—*b*y^2^ term:

10 $${\text{dy}}/{\text{dt}} = a{\text{y}} - b{\text{y}}^{2}$$

or in discrete form

11 $$\varDelta y_{\text{t}} \left( {{\text{or}}\,{\text{y}}_{{{\text{t}} + 1}} - {\text{y}}_{\text{t}} } \right) = a{\text{y}}_{\text{t}} - b{\text{y}}_{\text{t}}^{2} \quad (\varDelta {\text{t}} = 1)$$

This model is called *Logistic growth model* and is a *quadratic equation*. The graph of the function (placing x_t_ values in abscissa and derivates of the function in ordinate) is a parabola. As we said, it excludes an exponential, *ad infinitum* growth rate and describes a *bounded* system in its development implying a limit value (*carrying capacity*) beyond which the system no longer grows. In other terms, it reasonably expresses a limited growth process within the framework of a limited resource environment. So, the *y*^2^ term assures the *self*-*regulation* of the population if it gets too big. The restraint parameter *b* is a limiting rate expressing the set of factors that discourage the population growth. Generally, *b* will be very small compared to *a*, so that if *y* is not too large then the term—*b*y^2^ will be negligible compared to *a*y and the population will growth exponentially. If *y i*s very large, the term *b*y^2^ is no longer negligible, and thus serves to slow down the rapid rate of increase of the population. In this way, a feedback is introduced in system: population growth is now governed not only by a free growth mechanism but also by an adjustment mechanism competing with a free growth, whose action depends on the interaction between system state (population at a given time) and environmental resources. In other words, this interaction determines system’s *carrying capacity* (the maximum value that population can reach compatibly with available environmental resources). The presence of this second term end up destroying the linearity of growth law (Bertuglia and Vaio 2003, p. 128).

The nonlinear differential Logistic Eq. (10) (differential Logistic model in continuous times) has exact solutions whose trajectory or time trend is a S-shaped curve. The population *asymptotically* (that is, in the limit) approaches the straight line (the *carrying capacity*), either increasing or decreasing toward it depending on the initial population y_0_. The period of time before the population reaches half its limiting value is a period of accelerated growth and the solution curve rapidly increases. After this point, the rate of growth decreases and *in the long time* reaches zero. This is a period of diminishing growth and the solution curve gradually decelerates until it stabilizes (derivative set at zero). As it is known, analytically the exact solution is obtained by the following equation:

12 $$y(t) = \frac{{a{\text{y}}_{0} }}{{b{\text{y}}_{0} + \left( {a - b{\text{y}}_{0} } \right){\text{e}}^{{ - a({\text{t}} - {\text{t}}_{0} )}} }}$$

In the discrete case, if in Eq. (11) we divide by $\frac{a}{b}$—maximum level of sustainability—and, therefore, if we let x_t_ =  $\frac{yt}{{\frac{a}{b}}}$ or x_t_ =  $\frac{b}{a}$ y_t_, the *y* variable is transformed in the *x* variable (*x* values from 0 to 1), and we obtain the following difference logistic equation

13 $${\text{x}}_{{{\text{t}} + 1}} - {\text{x}}_{\text{t}} = a{\text{x}}_{\text{t}} - b{\text{x}}_{\text{t}}^{2} \quad (\varDelta {\text{t}} = 1,\;0 < x < 1)$$

14 $${\text{x}}_{{{\text{t}} + 1}} - {\text{x}}_{\text{t}} = a{\text{x}}_{\text{t}} \left( {1 - \frac{b}{a}{\text{x}}_{\text{t}} } \right)$$

and consequently its solution is

15 $${\text{x}}_{{{\text{t}} + 1}} = {\text{x}}_{\text{t}} + a{\text{x}}_{\text{t}} \left( {1 - \frac{b}{a}{\text{x}}_{\text{t}} } \right)$$

Trough several complex mathematical steps, it assumes the simplified structure of the *Logistic map* (*discrete Logistic equation*) (Bertuglia and Vaio 2003, p. 215). Indeed, if we let $\frac{b}{a}{\text{x}}_{\text{t}} = \frac{xt}{{\frac{a}{b}}}$ and $\frac{a}{b} = k$, we obtain

16 $${\text{x}}_{{{\text{t}} + 1}} = {\text{x}}_{\text{t}} + a{\text{x}}_{\text{t}} \left( {1 - \frac{xt}{k}} \right)$$

17 $${\text{x}}_{{{\text{t}} + 1}} = {\text{x}}_{\text{t}} + a{\text{x}}_{\text{t}} \left( {{\text{k}} - {\text{x}}_{\text{t}} } \right)$$

If we indicate the maximum limit *k* as L*k*, the (17) it can be rewritten as

18 $${\text{x}}_{\text{t + 1}} = {\text{x}}_{t} + a{\text{x}}_{t} \left( {{\text{L}}k - {\text{x}}_{t} } \right)$$

whose derivative equation

19 $${\text{x}}_{\text{t + 1}} - {\text{x}}_{\text{t}} = a{\text{x}}_{\text{t}} \left( {{\text{L}}k - {\text{x}}_{\text{t}} } \right)$$

is equivalent to (14).

By (18), if we let x_t_ =  $\frac{k(1 + a)}{a}$ x_t,_ we obtain

20 $$\frac{k(1 + a)}{a}{\text{x}}_{\text{t + 1}} = \frac{k(1 + a)}{a}{\text{x}}_{\text{t}} + a^{*}\frac{k(1 + a)}{a}{\text{x}}_{\text{t}}^{ *} \left( {1 - \frac{{\frac{k(1 + a)}{a}}}{k}{\text{x}}_{\text{t}} } \right),$$

and simplifying

21 $$\frac{k(1 + a)}{a}{\text{x}}_{\text{t + 1}} = \frac{k(1 + a)}{a}{\text{x}}_{\text{t}} + k\left( {1 + a} \right){\text{ x}}_{\text{t}} \left( {1 - \frac{1 + a}{a}{\text{x}}_{\text{t}} } \right)$$

If we multiply the (21) by $\frac{a}{k(1 + a)}$, the equation becomes

22 $${\text{x}}_{\text{t + 1}} = {\text{ x}}_{\text{t}} + a{\text{x}}_{\text{t}} \left( {1 - \frac{1 + a}{a}{\text{x}}_{\text{t}} } \right)$$

23 $${\text{x}}_{\text{t + 1}} = {\text{x}}_{\text{t}} + a{\text{x}}_{\text{t}} {-}\left( {1 + a} \right){\text{ x}}_{\text{t}}^{ 2}$$

24 $${\text{x}}_{\text{t + 1}} = \left( {1 + a} \right){\text{x}}_{\text{t}} {-}\left( {1 + a} \right){\text{x}}_{\text{t}}^{ 2}$$

Finally, if we let

$$\lambda = \left( {1 + a} \right)$$

we obtain the standard form of *Logistic map,* which in discrete time with Δt = 1 is the solution of differential Eq. (14):

25 $${\text{x}}_{\text{t + 1}} = \lambda {\text{x}}_{\text{t}} - \lambda {\text{x}}_{\text{t}}^{2} (\varDelta {\text{t}} = 1)$$

or else

26 $${\text{x}}_{\text{t + 1}} = \lambda {\text{x}}_{\text{t}} \left( {1 - {\text{x}}_{\text{t}} } \right)$$

Usually the notation λ is replaced with *k.* The *Logistic map*, x_t+1_ = *k* x_t_ (1 − x_t_), is the simplest nonlinear equation (of course, the nonlinearity regards the parameters). As well as in the continuous time equation, it is a *quadratic map* whose solution cannot generally be found using algebra but by numerical iteration. In more details, the equation indicates that x_t+1_ is a non-linear function of x_t_ (x_t+1_ = f(x_t_)). Analyzing in details the discrete equation, the formula tells us that the consistency of the x variable in t + 1 time, for example, of a biological population, or an organizational population (social groups like bureaucratic, political, industrial apparatus and so on) depends on the consistency of the variable over time t, according to a *k* growth parameter. This parameter is not constant as it would be if the growth were linear, because the environment cannot support unlimited growth. As we previously said, a certain population can reach a maximum number of individuals, according to the limited natural resources available. Thus, the more the population reaches its *carrying capacity* (maximum level of sustainability), the more the environment will discourage further growth. In other words, when population reaches the carrying capacity its growth is zero. This, however, it is not enough: the carrying capacity has to show its influence even before that population reaches its possible maximum, that is, it has to show its influence by restraining the speed or population growth rate in a degree as greater as x increases. So, the non-linear 1 − x_t_ component expresses the *restraint* that the environment poses on the x variable increase. The reasoning behind this is rather simple. In 1 − x_t_, 1 stands for 100 % environment carrying capacity, its theoretical limit of sustainability. Thus, if x_t_ were 1 % there would be 99 % resources left to sustain greater population increase (1–0.01), and 99 % multiplied by *k* (and then x_t_) can hardly lower the growth rate (if k were 2, the growth rate would be 99 % of 2, or 1.98 x_t_). On the contrary, if the population is 80 % of the maximum value sustainable by the environment, there will be few resources to sustain further growth or rather 20 % (1–0.80). The growth rate would be reduced by environment pressure (a 20 % of 2 reduction or 0.4 x_t_) and thus further growth would be discouraged. The population falls; hence, with low population values, growth still continues but at an ever decreasing rate, until *intime,* being growth rate more and more low, x_t_ stabilizes at a fixed value (if k = 2, x_t_ stabilizes at 0.50) (Marion 1999).

As observed by Marion, the whole question makes perfect logical sense, as well as naturally, mathematical sense (1999, p. 201).

The transformation from (11) to (13) equation is useful just because it “normalizes” the *y* variable between 0, the minimum value, and 1, the maximum value. This is particularly useful for the study of the model. It can be solved without considering the particular value of carrying capacity and simply expressing the population in percentage terms compared to the maximum allowed. This transformation imposes some constraints on *k* values: *k* cannot be negative (so that population does not become negative) and cannot exceed 4 (so that population does not exceed 1, which is the maximum allowed).

To find the equilibrium points of equation we let f (x*) = *k* x* (1 − x*) = x*. Thus, we pinpoint two equilibrium points (Elaydi 1991, p. 17):

$${\text{x}}^{*} = 0\quad {\text{and}}\quad {\text{x}}^{*} = \frac{k - 1}{k}\,\,{\text{or}}\,\,1 - \frac{1}{k}$$

The value of logistic approach become clear when one considers the type additional information it provides compared to traditional statistical approaches. Experts were fascinated by the surprisingly complex behavior by that which is the most simple of discrete non-linear one-dimensional systems. In effect, in spite of its simplicity, it exhibits a rather rich and complicated dynamics. The value of *k* describes the whole of ‘characteristic of the system’ which cause that system to be either stable, oscillating in a complex manner, or chaotic. So, if *k* = 0–3 the system stabilizes at *fixed point,* the so called *steady state,* representing system’s attractor. The graph of x_t_ values versus t shows a sequence of values that approaches a certain state and remain fixed there. For example, if *k* = 2 × variable reaches a balance between growth pressure and environmental constraints at $\frac{k - 1}{k}$ = 0.50 (Marion 1999). According to *k* value, all orbits (the succession of value x_0_, x_1_, x_2_,… starting from a specific value of x_0_), no matter the value of initial condition x_0_, tend to the same stable fixed point as t → ∞. In our example, all orbits, no matter where started, tend to the same stable fixed point of x = 0.50 as t → ∞. If growth rate *k* is less than 1, the population will get small until eventually it is infinitesimally small—for all practical purposes it ceases to exist. From 3 to 3.8 *k* values subsequent *bifurcations* (system change points) emerge and the equation describes a periodic behavior with ever increasing cycle lengths (2, 4, 8, 16, 32, 64, 128 etc. length cycles). The period doubles and the equation behavior exhibits a real *period*-*doubling cascade* or an infinite cycle sequence of period 2.^14^ The various cycles to which the process tend are the system’s attractors.^15^ This *bifurcation phenomenon* is widely acknowledged as the *road to chaos* (Feigenbaum 1978). When *k* is in the range of 3.8 to 4, the system’s behavior enters into a regime that Li and Yorke (1975) first named ‘chaotic’. Values are erratic, aperiodic, without ever repeating themselves, and therefore, appear to have no rules. The graph of the solutions of the nonlinear equation *versus* t shows that x_t_ values exhibit a random trend. *But randomness is merely apparent*, since the nature of mechanism or equation governing behavior is still deterministic. Hence the same mathematical model (the same equation) allows predictable behavior in some regions of model parameters, and unpredictable behavior in others. A chaotic change of x expresses an unpredictable asset of interactions among elements of the system (deviants, consumers social and political agents, atoms, ecological populations, etc.). As an example, if Logistic map is used for modeling volatility electoral, a chaotic behavior in the level of electoral support (number of votes) of a party expresses underlying disordered, instable, unpredictable political decision-making processes. Of course, it leads to reflect about the factors encouraging the predictability breakdown in the structure of political and social relationships (Brown 1991).

The chaotic behavior is the most interesting behavior which a simple, deterministic model such as Logistic map can exhibit. This is synthesized in the concept of *deterministicchaos,* expressing the new scientific idea according to which order and disorder are no longer opposing categories: disorder can come from order. This new acknowledge—this is the very interesting aspect—led to bridge the gap between scientific determinism and probabilism (Stewart 1989; for a more detailed description of structure and dynamical behavior of Logistic map see May 1976; Elaydi 1991; Kaplan and Glass 1995; Kostelich and Armbruster 1996; Condorelli 2007, 2013b).

**Fig. 1:**
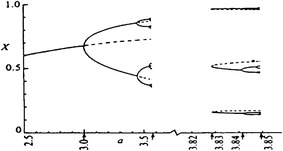
Stilization of bifurcation diagram or Feigenbaum tree (0 ≤ *x* ≤ , 0 ≤ *k* ≤)

In Fig. 1, below, we see clearly the system’s bifurcation points or the values of unstable equilibrium—placed on the ordinate—that *x* exhibits as a function of *k* value—placed on the abscissa:

We might say that *k* represents a sort of ‘regulating device’, when we turn it on we get a constant rise in dynamic behavior complexity: stationary → periodic → chaotic, with period doubling cascade as the mechanism generating chaos (Stewart 1989, p. 177).

It can be concluded that every phenomena whose process is modeled by the Logistic map expresses an underlying deterministic nature, which is responsible for every *bounded* evolution change including any eventual erratic behavior. In other words, the phenomena hides an underlying order which has its own formal and geometric ‘physiognomy’. As a rule, in the case of chaotic behavior, the underlying order takes on a bell-shaped attractor.

As we said, according to May, one of the major authorities in the study of dynamic behavior and logistic map, outside the physical sciences this non-linear model is *the rule* and *not the exception* (May 1976, p. 467). In effect, its use in the Social Sciences is highly pervasive. Every time a social phenomenon has been studied from a possible non-linear perspective, the Logistic map—just because it is able to include regions of predictable behavior, regions of chaos and transitions between such regions—appeared to be the most suitable for modeling its behavior and for reconstructing its underlying governing mechanism. As we said, this is precisely what happened in the study of social phenomena as population evolution, price analysis, political competition (Brown 1991), arms race between nations (Saperstein, 1984, 1997a, b; Grossmann and Mayer-Kress 1989; Campbell and Mayer-Kress 1997), drug use (Priesmeyer 1995), spread of new technologies and informations (West 1997), crime and infant mortality (Priesmeyer 1995; Huckfeldt 1989; Condorelli 2013c).

The discovery of this *regularity* is of relevant consequence for the Social Sciences. We can say that the Logistic map *is* the structure of a non-linearly oriented social phenomenon. We can consider social phenomena as not being susceptible to unlimited growth but rather *bounded* in their development. In short, factors which contribute to the growth and factors whose action reduces their growth rate, *taken collectively*, provides the limits of a *bounded* social system, thereby preventing an exponential increase. The *k*-value in social system provides important information about intervention and control processes. Low *k*-values suggests bounded process, far from a chaotic behavior, being able either to increase or decrease depending upon the current state of the system. When system’s behavior flows into a chaotic behavior every predictability and control capacity is lost.

### Anatomy of suicide: mathematical formalization of suicide decision-making process by a nonlinear Logistic model

From our perspective, the appropriateness to model suicide growth by a difference Logistic equation is based on the following theoretical reasoning about the structure of suicide decision-making process integrating Durkheim’s Theory with Halbwachs’s Theory:

Why do some people suicide? More importantly, why do some people suicide and why some people do not commit suicide? On the hand, from a durkheimian point of view weakening of social ties, social isolation due to individualism and modernization processes is the reason inducing a subject to suicide and therefore it determines the suicide growth rate (*a*) On the other hand, we can assume, as Halbwachs said, that suicides can exist within a maximum limit of social sustainability. Thus they can spread but only up to a certain point, beyond which the suicide cannot go. This maximum growth limit is defined here as L_S_ (*carrying capacity*): the maximum proportion of citizens who *might* commit suicide. Factors which contribute to the growth (social fragmentation as well as the lack of material prosperity in adverse economic conditions) and factors able to restrain the suicide growth rate by encouraging an adptation process to modernization stresses (internalization of ethic-religious value of life, material benefits of industrial and economic progress being able to compensate weakening of social ties, social services being able to accommodate modern person (i.e. aged and women) and create a less suicidogenic environment, and mainly the emergence of a consumption-oriented mentality and a new cultural pattern far from traditional, familiar and religious, values), *taken collectively,* provide the limits of a *bounded* social system. In particular, factors whose action *restrains* suicide growth rate fix the width of adaptation sphere (1 − L_S_) or the proportion of people who would not suicide due to the cited factors and adapt to social fragmentation. Therefore, given the proportion of citizens who suicides at time t, it follows that the proportion of citizens eligible to commit suicide at the next time t + 1 is equal to L_S_ − S_t_: the proportion of suicides which *might* be committed due to factors encouraging it. So, suicide growth rate is influenced from adaptation sphere, that is, the pool of potential suicides depends on the width of this sphere. The wider the (1 − L_S_) sphere is, the narrower the space for potential suicides (L_S_ − S_t_), and the lower the limits of growth and expansion of suicide. On the contrary, the more (1 − L_S_) is restricted, larger the number of potential suicides (L_S_ − S_t_). Thus L_S_ is defined as being fixed in time, while S_t_ varies through time. Before the maximum growth limit, that is, up to that maximum diffusion of suicides (Ls), the more the level S_t_ of suicides increases, the more the proportion of people who *might* suicide due to the weakening of social cohesion (L_S_ − S_t_) will become small more and more, so that suicide growth rate *a* is restrained and a further growth is discouraged.

According to these considerations, we can build a model able to describe suicide growth rate trend (s_t+1_) by a logistic structure on the rate of growth (difference logistic Eqs. 13, 14, 19):

$${\text{S}}_{\text{t + 1}} - {\text{S}}_{\text{t}} \, \left( {{\text{or}}\,{\text{rather}}\,{\text{s}}_{\text{t + 1}} } \right) = {\text{S}}_{\text{t}} - b{\text{S}}_{\text{t}}^{2}$$

$${\text{S}}_{\text{t + 1}} - {\text{S}}_{\text{t}} = a{\text{S}}_{\text{t}} ({\text{L}}_{\text{S}} - {\text{S}}_{\text{t}} )$$

Its solution is

27 $${\text{S}}_{\text{t + 1}} = {\text{S}}_{\text{t}} + a{\text{S}}_{\text{t}} ({\text{L}}_{\text{S}} - {\text{S}}_{\text{t}} )\quad (\varDelta {\text{t}} = 1)$$

or rather

$${\text{S}}_{\text{t + 1}} = k{\text{S}}_{\text{t}} ({\text{L}}_{\text{S}} - {\text{S}}_{\text{t}} )\quad k = 1 + a \quad \left( {1 + {\text{non}}\,{\text{constant}}\,{\text{percentage}}\,{\text{of}}\,{\text{growth}}} \right)$$

Suicide population level at time t + 1 (as integration of suicide difference equation) depends from suicide population level in time t (S_t_) according to a rate of change *k* that is not constant. *k,* synthesizing the impact of factors that would induce a subject to suicide (especially the social fragmentation and isolation process), varies depending on the maximum limit that suicide population can reach or *carrying capacity* Ls, and therefore on the number of people eligible for suicide L_S_ − S_t_, (suicide number which could be potentially committed). By normalizing the suicide variable between 0, the minimum value, and 1, the maximum value, the Eq. (27) becomes equivalent to

28 $${\text{S}}_{\text{t + 1}} = k{\text{S}}_{\text{t}} \;(1 - {\text{S}}_{\text{t}} )$$

As we previously said, 1 is the maximum of social sustainability being allowed (100 %). The more the consistency S_t_ of actual suicides increases and reaches its possible maximum level of social sustainability the less people (1 − S_t_) remain which *might* commit suicide limiting the rate of growth. S_t+1_ varies as nonlinear function of (Ls − S_t_) or rather (1 − S_t_). According to *k* value, suicide behaviour can range from steady states (regions of predictable behaviour) to chaos (regions of erratic behaviour and predictability breakdown).

### Application of the Logistic model to suicide data in Italy: methodological aspects

**Fig. 2:**
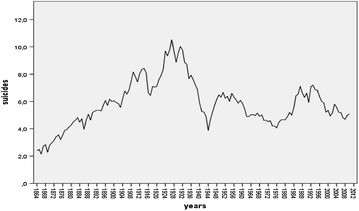
Suicide rates in Italy—1864–2010. Elaboration from ISTAT Source—Suicidi e Tentativi di Suicidio—1864–2010

As we said in the present research paper we attempt to model the structure of suicide decision-making process and to verify whether Logistic model is adapt in order to capture longitudinal change in suicides levels in a body of real data, namely Italian suicide from 1864 to 2010. We elaborated suicide rates by using suicide data published from ISTAT-*Statistics Italian Institute*—1864–2010 (each annual number of observed suicides was divided by total Italian population in the considered span of time and multiplied per 100,000; Fig. 2).

Among the most industrialized OECD countries Italy has one of the lowest levels of suicide mortality (Table 1 in Appendix 1). As Table 1 shows, from 1993 to 2010 (the latest available year) ISTAT reported a suicide rate decrease from 7.3 to 5.0 suicides per 100,000 inhabitants, with variations at the lowest historical levels in recent years (ISTAT 2012a, b). The Table shows the same time trend for all OECD countries.

**Table 1:** Suicide mortality: some international comparisons-suicide rates per 100.000 inhabitants 1993–2010

	1993	1994	1995	1996	1997	1998	1999	2000		
Austria	21.4	22.2	22.1	22.0	19.4	19.2	18.9	19.4		
Belgium	21.3	21.0	21.1	19.6	20.9	19.6	17.8	–		
Denmark	22.1	18.9	17.5	16.8	15.4	14.2	14.2	13.5		
Finland	27.4	27.2	26.9	24.0	25.5	23.5	23.1	22.1		
France	21.6	21.0	20.4	19.4	19.0	18.0	17.5	18.2		
Germany	15.2	15.2	15.3	14.4	14.4	13.6	12.9	12.8		
Greece	4.0	3.4	3.5	3.3	3.4	3.6	3.4	3.4		
Ireland	10.0	11.9	11.8	11.6	13.1	13.7	11.6	12.3		
Iceland	10.9	10.2	10.7	13.5	13.7	11.3	11.4	18.1		
Italy (a)	8.3	8.1	8.1	8.3	8.3	8.0	7.3	7.3		
Italy (b)	7.3	7.0	6.9	6.5	6.1	6.0	5.3	5.5		
Norway	13.7	12.4	12.7	11.8	12.3	12.5	13.4	12.3		
Netherlands	10.3	10.4	9.8	10.2	10.1	9.6	9.6	9.4		
Portugal	8.1	7.9	8.2	6.7	6.2	5.4	5.3	5.0		
UK	7.7	7.5	7.4	7.1	7.0	7.4	7.5	–		
Spain	8.0	8.2	8.0	8.4	8.4	8.0	7.8	8.1		
Sweden	15.6	14.8	15.1	13.9	13.4	13.6	13.5	12.4		
Switzerland	20.2	21.3	20.1	20.1	18.7	19.0	17.7	18.7		
Australia	11.9	13.1	12.2	13.5	14.5	14.1	13.3	12.6		
Canada	13.3	13.0	13.4	13.3	12.3	12.2	13.2	11.5		
USA	12.6	12.5	12.4	12.1	11.9	11.7	11.1	10.8		

	2001	2002	2003	2004	2005	2006	2007	2008	2009	2010
Austria	18.0	18.6	17.4	16.7	16.2	14.7	14.5	14.1	14.1	13.9
Belgium	–	–	–	18.4	18.6	–	–	–	–	–
Denmark	13.3	12.5	11.5	12.0	11.3	11.6	–	–	–	–
Finland	22.8	20.7	20.1	20.0	18.3	19.6	18.2	19.0	18.9	17.3
France	17.5	17.6	17.8	17.5	17.1	16.5	15.8	16.1	–	–
Germany	12.8	12.7	12.6	12.0	11.4	10.7	10.2	10.3	10.3	10.8
Greece	2.9	2.8	3.3	3.0	3.4	3.3	2.8	3.1	3.2	–
Ireland	12.8	11.4	11.5	11.5	10.8	10.6	10.4	11.5	11.7	11.0
Iceland	12.8	10.3	9.8	12.5	11.5	10.8	12.0	12.4	11.8	–
Italy (a)	7.1	7.2	7.1	–	–	6.3	6.4	6.6	6.7	–
Italy (b)	5.0	5.2	5.9	5.7	5.0	5.2	4.9	4.7	5.0	5.10
Norway	12.4	11.0	11.1	11.8	11.6	11.5	10.5	10.6	11.9	11.2
Netherlands	9.1	9.6	9.2	9.1	9.4	9.1	8.0	8.4	8.9	9.2
Portugal	7.2	11.3	10.6	–	–	–	8.9	9.0	8.9	9.3
UK	7.0	6.9	6.6	6.9	6.7	6.7	6.3	6.9	6.8	6.7
Spain	7.4	7.7	7.8	7.7	7.3	6.9	6.7	7.0	6.9	–
Sweden	13.0	12.9	12.0	12.4	13.1	12.7	11.9	12.2	12.9	11.7
Switzerland	18.0	19.0	16.6	16.6	16.6	16.5	16.9	–	–	–
Australia	12.8	11.9	10.9	10.6	–	8.3	–	–	–	–
Canada	11.7	11.5	11.7	11.0	11.2	10.5	10.6	10.7	11.1	–
USA	11.1	11.3	11.1	11.3	11.2	11.3	11.7	–	–	–

*Source*: OECD (Organization for Economic Cooperation and Development)

Italy (a) Source: ISTAT—*Decessi e Cause di morte*

Italy (b) Source: ISTAT—*Suicidi e Tentativi di suicidio*. Judicial Statistics do not include suicides in prison and suicide cases in which the death occurs after a few days from the episode that actually caused the death (such cases are recorded as “attempted suicide” cases)

**Fig. 3:**
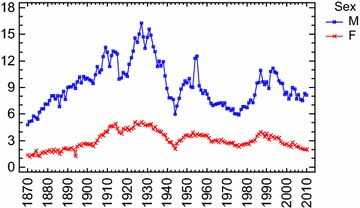
Suicide rates by sex—Italy—1864–2010

**Fig. 4:**
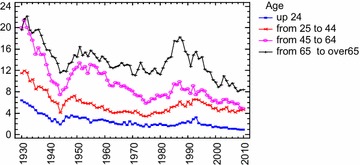
Suicide rates by age—Italy—1928–2010

**Fig. 5:**
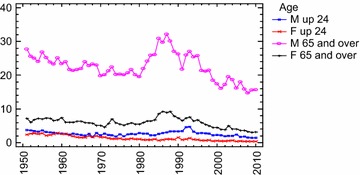
Suicide rates by sex and age (0–24, 65 and over)—Italy—1950–2010

**Fig. 6:**
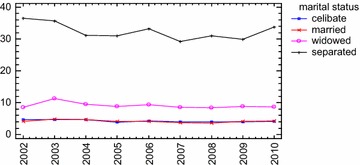
Suicide rates by marital status—2000–2010

Statistics show a regularity in reference to main at risk social categories, confirming the Durkheim’s acquisitions. The propensity to suicide is higher among men (men suicide more than women independent of age) and increases as age increases (Figs. 3, 4, 5). Being married is a protective factor for suicide: divorced/widowed/separated suicide more than married (Fig. 6), and divorced/widowed/separated men suicide more than divorced/widowed/separated women (EURES 2012).

This being stated, our analysis divides in two step.

### First step

First, we analyzed suicide time trends by applying the *Bayesian Change Point Analysis* to overall suicide rates in order to identify the years in which time trend changes *significantly.*

The analysis has confirmed the findings we found in our previously suicide rate analysis until 2005, by identifying 5 change points: 1876–1893–1961–1984–1996, *p* (*r*_1_*, r*_2_, *r*_3_, *r*_4_, *r*_5_) = 0.0324 (considering that with 5 change points the number of obtainable combinations exceeds the million and most posterior probability *p* (*r*) is almost null, a 3 % posterior probability constitutes a significant value). 1876–1961 and 1996 continue to be the most important change points: beginning from 1876, after the first industrial development, suicide rates increase; however, beginning from 1961, in connection to the Italian *economic and industrial boom*, suicide rates decrease, and they do not increase as expected from a Durkheimian theoretical perspective; after increasing from 1984 to 1996, they continue to decrease from 1996 to 2010. Anyway, they no longer reach the maximum levels attained between the two wars (for a detailed description of change point technique see Condorelli 1998, 2013a).

### Second step

The Italian suicide rate time series (s_t*i*_) can be thought of as the derivative or rather the growth rate (s_t*i*_ = S_t*i* +1_ − S_t*i*_, with *i* from 0 to N) of a change process in the level of Suicide Population (S_t*i*_) which is modeled by a nonlinear logistic difference equation such as the 3.10 equation whose solution (S_t*i*_) is the Logistic map 3.17.2. If we look at the Fig. 2, 3, 4, 5, 6, the physical mechanism of suicide growth rate seems far from the one of derivates of a linear growth process (in this case the plot of derivative exhibits a flat line) or of an exponential process (rising or descending straight line). Instead, it seems to approximate the physical mechanism of derivates of a restrained or Logistic growth process exhibiting a parabolic trend and describing the change process of a *bounded* system which evolves incrementally from the first year forward. Consequently, starting from 1873 and fixing the initial condition in 1875 just after the industrial *feverish triennium, w*e estimated the Logistic map for observed integrative suicide population data St_1874_ = s_t1873_ + s_t1874_, St_1875_ = S_t1874_ + s_t1875_, S_t1876_ = St_1875_ + s_t1876…_, S_tn_ = ∑s_ti_*i* = 0–N, N = 136) of which each suicide rate (s_t*i*_) of the observed time series can be considered the derivative or growth rate. For this purpose we followed the procedure which was suggested from Priesmeyer (1995) in order to find the best estimate of model’s parameters and the best fit to data. First, because the Logistic model is nonlinear in parameters, solutions were computed by iterating the Logistic map 3.17.2 by taking different values of parameter k and initial condition x, with each subsequent iteration being built upon the results of the previous one (unlike a linear regression equation which fits data to an arbitrary independent variable by a sequential count or year; Priesmeyer 1995, p. 335). Second, depending on the initial values of the parameters, from time to time the fit was measured between standardized predicted logistics values and standardized suicide population data.^16^ As we said, the model is evaluated on its ability to explain longitudinal change in suicide levels for the period in consideration, detecting *how* the system changes and revealing underlying systemic patterns from within the data. Therefore, the model for which the sum of the squares of the difference among standardized ‘ideal’ predicted values and standardized actual values was minimized represented the best fit to data (that is, it presented the best estimate of parameters). Following are the steps taken to estimate k and initial condition x in order to fit the model to data set (Priesmeyer 1995, p. 337–338).

#### Step 1

Standardize the target data (Ts) by the equation

$${\text{Zn}} = \frac{{{\text{Xn }}{-}{\text{M}}}}{\sigma },\quad {\text{where }}\sigma \left( {\text{standard deviation}} \right) = \frac{{\sum \left( {{\text{x}} - {\text{M}}} \right)^{2} }}{\text{n}}$$

#### Step 2

Compute repeated series of logistic values with n (n = 147) observations by using the formule

$${\text{x}}_{\text{t+1}} ={\rm k}{\text{x}}_{\text{t}} \left( {1 - {\text{x}}_{\text{t}} } \right)$$

Iterate Logistic map starting from different initial values of x and k parameter, with each subsequent iteration being built upon the result of the previous. Increment x within increments of k. k ranges from 0 to 4 while x ranges from 0 to 1. Step the incrementing of k and x by 0.01, or smaller.

#### Step 3

Standardize each series of logistic values computed in Step1.

#### Step 4

Compute R2 as measure of the quality of fit between the standardized target (Ts) and standardized logistic values (predicted Ts or Tp). The equation is

$${\text{R}}^{2} = \frac{{\sum ({\text{Ts}}-\overline{\text{Ts}})^{2}}}{{\sum ({\text{Ts}} - \overline{\text{Ts}})^{2}}} - \sum \left({{\text{Ts}} - {\text{Tp}}} \right)^{2}$$

where $\sum ({\text{Ts}} - \overline{\text{Ts}} )^{2}$ is the sum of squares total, ∑ (Ts − Tp)^2^ is the sum of squares error (difference between standardized target and logistic predicted values), and ∑ (Ts −  $\overline{\text{Ts}}$)^2^ − ∑ (Ts − Tp)^2^ is the predicted deviance. Because the mean $\overline{\text{Ts}}$ of Ts is 0, the formule becomes

$${\text{R}}^{2} = \frac{{\sum {\text{Ts}}^{2} - \sum \left( {{\text{Ts}} - {\text{Tp}}} \right)^{2} }}{{\sum {\text{Ts}}^{2} }}$$

The model with the values of k and x which minimize the sum squares error, maximizing predicted deviance and therefore R^2^ values, is the model that best fits the observed data.

#### Step 5

Compute the fitted measures F from an iterative logistic by using the following expression:

$${\text{F}} = {\text{u}}_{t} + \left({{\text{kx}}_{t}\left({1 - {\text{x}}_{\text{t}}} \right) - {\text{u}}_{\text{j}}} \right)\times \left({\text{ds}_{t}}/{ \text{ds}}_{j} \right)$$

F = fitted estimate for each observation, x_t+1_ = kx_t_ (1 − x_t_) = logistic values, u_t_ = mean of the target values, u_j_ = mean of the logistic values, ds_t_ = standard deviation of target values, ds_j_ = standard deviation of logistic values

Finally, based on the estimate of the model that best fits the observed data St, the derivative st was computed by the corresponding nonlinear difference equation; fitted suicide growth rates was computed and compared to observed Italian suicide growth rates.

## Results and discussion

The analysis found the highest R^2^ among standardized target data (Mean and Standard Deviation of target data was: M = 427.75, SD = 252.88) and standardized logistic values at 1.065 *k*-value and initial condition 0.001983. Nonlinear Logistic map explained 96 % of the variance in Italian suicide population S_t_ (R^2^ = 0.95):

**Table:** 

Suicides 1875–2010	Obs.	*k*	x	R^2^	M	SD
Total	136	1.07	0.001983	*0.96*	0.03685	0.02211

According to these findings, low k-value of 1.065 represents a tightly bounded growth process within the social fabric *far from* the chaotic behavior. As we said, a 3.8–4 k values suggests an social scenario in which social interactions system is instable, and it is so sensitive to initial conditions that any action aiming to change the incidence of the system any way produces unpredictable results. Priesmayer provided us with an exemplifying case, regarding cocaine use in the USA from 1985 to 1990. In his investigation, the logistic equation fitted cocaine use with a value *k* of 3.6, dangerously close to the critical threshold of 3.8, a value inducing the author to the following disarming conclusion:

Put simply, actions which decrease current use may contribute to higher future use or they may not; actions which contribute to higher current use may contribute to lowering use in the future or they may not. […] Does it suggest that […]attempts to lower cocaine use by aggressive intervention are far less certain? If cocaine use is not controllable in this way, what then is to be used to control cocaine use? (Priesmayer 1990, p. 333).

Regarding suicide in Italy the situation seems to be different. In this case, low *k* value suggests that social system, as a *whole,* is able to self-organizing and adapt spontaneously to modernization effects showing a restrained suicide growth process and remaining *far from maximum entropy or disorder* (far from loss of control and predictability).

**Fig. 7:**
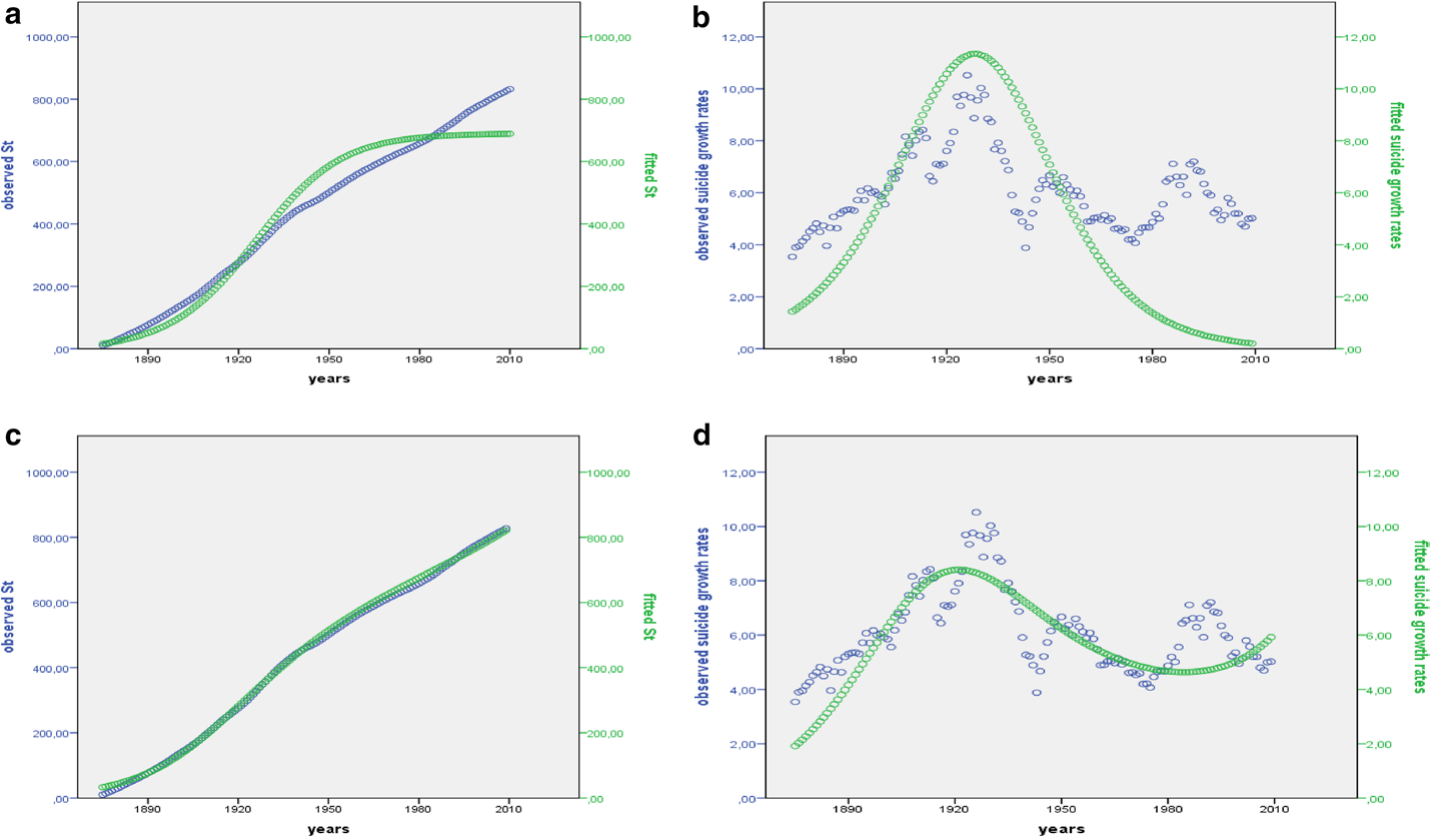
**a** Observed Suicide population St and Fitted Suicide population that was estimated by logistic map with initial condition 0,001983. **b** Italian suicide growth rates and Fitted suicide growth rates (s_t_ or derivates) that was estimated by logistic model with initial condition of 0.001983. **c** Observed Suicide population St and Fitted Suicide population that was estimated by the model (36) with initial condition 0.0030. **d** Italian suicide growth rates and Fitted suicide growth rates (s_t_ or derivatives) that was estimated by the model (33) with initial condition of 0.0030

However, still more in details, although *on the whole* the fit among standardized target data and standardized logistic values appears high, at 96 % (the same result was obtained by computing R^2^ among actual and fitted S_t_ data too), and fitted suicide growth rates reproduce the rapid increase of actual suicide growth rates until 1927–1930 (with Fascism as possible contributing factor^17^) and their subsequent decrease until the 60 s and 70 s (Table 2 in Appendix 2; Fig. 7a, b), the percentage difference between fitted and actual data seems to suggest the usefulness to improve the model in order to reduce the residual variance. In particular, coinciding with Italian monetary and financial crisis of 80 and 90 s, actual suicide population S_t_ and actual suicide growth rates s_t_ increase more than fitted data. The growth is restrained but suicide growth rates s_t_ asymptotically do not tend to zero such as the model implies. Consequently, suicide population S_t_ continues to increase even though it shows a growth rate restrained compared to the past growth. Therefore, in order to improve the model we added to the quadratic, logistic growth a cubic component, which could be susceptible to model the rising trend, although restrained, in suicides in the considered years. This is in line with our assumptions: our hypothesis concedes that, if in the long run the effects of individualization process can be offset by the benefits of industrial progress, for the same reasons situations of economic crisis may have a positive impact on suicide decision-making. To model suicide growth rate taking into account this possibility, we used the following differential model having logistic structure (quadratic structure in its first part, Eq. 13):

29 $${\text{x}}_{\text{t + 1}} - {\text{x}}_{\text{t}} = a{\text{x}}_{\text{t}} - b{\text{x}}_{\text{t}}^{ 2} + c{\text{x}}_{\text{t}}^{3}$$

whose solution is

30 $${\text{x}}_{\text{t + 1}} = {\text{x}}_{\text{t}} + (a{\text{x}}_{\text{t}} - b{\text{x}}_{\text{t}}^{ 2} + c{\text{x}}_{\text{t}}^{3} )$$

or else

31 $${\text{x}}_{\text{t + 1}} = \left( {1 + a} \right){\text{ x}}_{\text{t}} {-}b{\text{x}}_{\text{t}}^{2} + c{\text{x}}_{\text{t}}^{3}$$

and again

32 $${\text{x}}_{\text{t + 1}} = {\text{ k}}_{ 1} {\text{x}}_{\text{t}} + {\text{ k}}_{ 2} {\text{x}}_{\text{t}}^{2} + {\text{k}}_{ 3} {\text{x}}_{\text{t}}^{3}$$

with

$${\text{k}}_{1} = 1 + a,{\text{ k}}_{2} = b,{\text{ k}}_{ 3} = c$$

**Table 2:** Observed and fitted data by the model S_t+1_ = 1.065 × 0.0019830 × (1–0.0019830)

a	b (1)	b (2)	b (2)	c	d	e	f	g (1)	g (2)	h
Years	Observed suicide growth rates s_t_	Observed S_t_	Observed suicide growth rates s_t_ (1876–1875. etc.)	Z obs. S_t_ (**b** **2**)	Predicted S_t_	Z predic. S_t_	Fitted St	Residues **b(2)** − **f** from 1875	% Residues (**f/b(2)**) × 100	Fitted derivative or suicide growth rates S_t+1_ − S_t_
R^2^ = 0.96										
1873	3.43									
1874	3.55									
1875	3.21	10.19		−1.65	0.003000	−1.56	33.26	−23.07	−226.37	
1876	3.54	13.73	3.54	−1.64	0.003192	−1.55	35.20	−21.47	−156.37	1.94
1877	3.90	17.63	3.90	−1.62	0.003395	−1.54	37.25	−19.62	−111.30	2.05
1878	3.95	21.58	3.95	−1.61	0.003610	−1.54	39.43	−17.85	−82.70	2.18
1879	4.14	25.72	4.14	−1.59	0.003837	−1.53	41.72	−16.00	−62.22	2.29
1880	4.27	29.99	4.27	−1.57	0.004077	−1.52	44.15	−14.16	−47.22	2.43
1881	4.51	34.50	4.51	−1.56	0.004330	−1.51	46.71	−12.21	−35.39	2.56
1882	4.63	39.13	4.63	−1.54	0.004597	−1.50	49.41	−10.28	−26.27	2.70
1883	4.82	43.95	4.82	−1.52	0.004879	−1.48	52.26	−8.31	−18.92	2.85
1884	4.49	48.44	4.49	−1.50	0.005176	−1.47	55.27	−6.83	−14.10	3.01
1885	4.74	53.18	4.74	−1.48	0.005489	−1.46	58.43	−5.25	−9.88	3.16
1886	3.96	57.14	3.96	−1.47	0.005818	−1.45	61.76	−4.62	−8.09	3.33
1887	4.65	61.79	4.65	−1.45	0.006163	−1.43	65.25	−3.46	−5.60	3.49
1888	5.07	66.86	5.07	−1.43	0.006525	−1.42	68.91	−2.05	−3.07	3.66
1889	4.63	71.49	4.63	−1.41	0.006905	−1.40	72.76	−1.27	−1.77	3.85
1890	5.20	76.69	5.20	−1.39	0.007303	−1.39	76.78	−0.09	−0.12	4.02
1891	5.30	81.99	5.30	−1.37	0.007720	−1.37	81.00	0.99	1.21	4.22
1892	5.35	87.34	5.35	−1.35	0.008155	−1.35	85.40	1.94	2.22	4.40
1893	5.36	92.70	5.36	−1.32	0.008609	−1.34	89.99	2.71	2.92	4.59
1894	5.31	98.01	5.31	−1.30	0.009083	−1.32	94.79	3.22	3.29	4.80
1895	5.72	103.73	5.72	−1.28	0.009576	−1.30	99.77	3.96	3.81	4.98
1896	6.07	109.80	6.07	−1.26	0.010089	−1.28	104.96	4.84	4.40	5.19
1897	5.71	115.51	5.71	−1.23	0.010622	−1.26	110.36	5.15	4.46	5.40
1898	6.17	121.68	6.17	−1.21	0.011174	−1.23	115.94	5.74	4.72	5.58
1899	6.00	127.68	6.00	−1.19	0.011746	−1.21	121.72	5.96	4.66	5.78
1900	6.05	133.73	6.05	−1.16	0.012338	−1.19	127.71	6.02	4.50	5.99
1901	5.92	139.65	5.92	−1.14	0.012949	−1.16	133.89	5.76	4.12	6.18
1902	5.85	145.50	5.85	−1.12	0.013579	−1.14	140.27	5.23	3.60	6.38
1903	5.56	151.06	5.56	−1.09	0.014227	−1.11	146.82	4.24	2.81	6.55
1904	6.18	157.24	6.18	−1.07	0.014893	−1.08	153.56	3.68	2.34	6.74
1905	6.77	164.01	6.77	−1.04	0.015578	−1.06	160.49	3.52	2.15	6.93
1906	6.54	170.55	6.54	−1.02	0.016278	−1.03	167.57	2.98	1.75	7.08
1907	6.84	177.39	6.84	−0.99	0.016996	−1.00	174.83	2.56	1.44	7.26
1908	7.47	184.86	7.47	−0.96	0.017728	−0.97	182.23	2.63	1.42	7.40
1909	8.16	193.02	8.16	−0.93	0.018475	−0.94	189.79	3.23	1.67	7.56
1910	7.83	200.85	7.83	−0.90	0.019236	−0.91	197.49	3.36	1.67	7.70
1911	7.43	208.28	7.43	−0.87	0.020009	−0.88	205.31	2.97	1.43	7.82
1912	8.02	216.30	8.02	−0.84	0.020794	−0.85	213.25	3.05	1.41	7.94
1913	8.34	224.64	8.34	−0.80	0.021590	−0.82	221.30	3.34	1.49	8.05
1914	8.42	233.06	8.42	−0.77	0.022394	−0.78	229.43	3.63	1.56	8.13
1915	8.10	241.16	8.10	−0.74	0.023208	−0.75	237.67	3.49	1.45	8.24
1916	6.64	247.80	6.64	−0.71	0.024028	−0.72	245.96	1.84	0.74	8.29
1917	6.44	254.24	6.44	−0.69	0.024855	−0.69	254.32	−0.08	−0.03	8.36
1918	7.10	261.34	7.10	−0.66	0.025686	−0.65	262.73	−1.39	−0.53	8.41
1919	7.05	268.39	7.05	−0.63	0.026522	−0.62	271.19	−2.80	−1.04	8.46
1920	7.11	275.50	7.11	−0.60	0.027360	−0.59	279.66	−4.16	−1.51	8.47
1921	7.61	283.11	7.61	−0.57	0.028200	−0.55	288.16	−5.05	−1.78	8.50
1922	7.91	291.02	7.91	−0.54	0.029040	−0.52	296.66	−5.64	−1.94	8.50
1923	8.34	299.36	8.34	−0.51	0.029880	−0.48	305.15	−5.79	−1.94	8.49
1924	9.69	309.05	9.69	−0.47	0.030719	−0.45	313.64	−4.59	−1.49	8.49
1925	9.34	318.39	9.34	−0.43	0.031555	−0.42	322.10	−3.71	−1.16	8.46
1926	9.76	328.15	9.76	−0.39	0.032388	−0.38	330.52	−2.37	−0.72	8.42
1927	10.52	338.67	10.52	−0.35	0.033217	−0.35	338.91	−0.24	−0.07	8.39
1928	9.67	348.34	9.67	−0.31	0.034042	−0.32	347.25	1.09	0.31	8.34
1929	8.87	357.21	8.87	−0.28	0.034861	−0.29	355.54	1.67	0.47	8.29
1930	9.55	366.76	9.55	−0.24	0.035674	−0.25	363.76	3.00	0.82	8.22
1931	10.03	376.79	10.03	−0.20	0.036480	−0.22	371.91	4.88	1.29	8.15
1932	9.76	386.55	9.76	−0.16	0.037279	−0.19	380.00	6.55	1.70	8.09
1933	8.85	395.40	8.85	−0.13	0.038070	−0.16	388.00	7.40	1.87	8.00
1934	8.72	404.12	8.72	−0.09	0.038853	−0.13	395.92	8.20	2.03	7.92
1935	7.67	411.79	7.67	−0.06	0.039628	−0.09	403.76	8.03	1.95	7.84
1936	7.92	419.71	7.92	−0.03	0.040394	−0.06	411.50	8.21	1.95	7.74
1937	7.61	427.32	7.61	0.00	0.041150	−0.03	419.15	8.17	1.91	7.65
1938	7.22	434.54	7.22	0.03	0.041898	0.00	426.72	7.82	1.80	7.57
1939	6.87	441.41	6.87	0.05	0.042636	0.03	434.18	7.23	1.64	7.46
1940	5.91	447.32	5.91	0.08	0.043365	0.05	441.56	5.76	1.29	7.38
1941	5.27	452.59	5.27	0.10	0.044084	0.08	448.83	3.76	0.83	7.27
1942	5.22	457.81	5.22	0.12	0.044793	0.11	456.00	1.81	0.39	7.17
1943	4.89	462.70	4.89	0.14	0.045492	0.14	463.07	−0.37	−0.08	7.07
1944	3.88	466.58	3.88	0.15	0.046182	0.17	470.05	−3.47	−0.74	6.98
1945	4.67	471.25	4.67	0.17	0.046863	0.19	476.94	−5.69	−1.21	6.89
1946	5.21	476.46	5.21	0.19	0.047533	0.22	483.72	−7.26	−1.52	6.78
1947	5.73	482.19	5.73	0.22	0.048194	0.25	490.40	−8.21	−1.70	6.68
1948	6.15	488.34	6.15	0.24	0.048846	0.27	497.00	−8.66	−1.77	6.60
1949	6.48	494.82	6.48	0.27	0.049488	0.30	503.49	−8.67	−1.75	6.49
1950	6.32	501.14	6.32	0.29	0.050122	0.32	509.91	−8.77	−1.75	6.42
1951	6.67	507.81	6.67	0.32	0.050746	0.35	516.22	−8.41	−1.66	6.31
1952	6.24	514.05	6.24	0.34	0.051362	0.37	522.45	−8.40	−1.63	6.23
1953	6.37	520.42	6.37	0.37	0.051969	0.40	528.59	−8.17	−1.57	6.14
1954	6.00	526.42	6.00	0.39	0.052568	0.42	534.65	−8.23	−1.56	6.06
1955	6.60	533.02	6.60	0.42	0.053158	0.45	540.62	−7.60	−1.42	5.97
1956	6.31	539.33	6.31	0.44	0.053741	0.47	546.51	−7.18	−1.33	5.89
1957	6.12	545.45	6.12	0.47	0.054316	0.49	552.33	−6.88	−1.26	5.82
1958	5.88	551.33	5.88	0.49	0.054883	0.52	558.06	−6.73	−1.22	5.73
1959	6.08	557.41	6.08	0.51	0.055444	0.54	563.74	−6.33	−1.14	5.68
1960	5.86	563.27	5.86	0.54	0.055997	0.56	569.33	−6.06	−1.08	5.59
1961	5.48	568.75	5.48	0.56	0.056544	0.58	574.87	−6.12	−1.08	5.54
1962	4.89	573.64	4.89	0.58	0.057084	0.60	580.33	−6.69	−1.17	5.46
1963	4.90	578.54	4.90	0.60	0.057618	0.62	585.73	−7.19	−1.24	5.40
1964	5.03	583.57	5.03	0.62	0.058146	0.65	591.07	−7.50	−1.29	5.34
1965	5.05	588.62	5.05	0.64	0.058668	0.67	596.35	−7.73	−1.31	5.28
1966	4.97	593.59	4.97	0.66	0.059185	0.69	601.58	−7.99	−1.35	5.23
1967	5.14	598.73	5.14	0.68	0.059696	0.71	606.75	−8.02	−1.34	5.17
1968	4.92	603.65	4.92	0.70	0.060203	0.73	611.88	−8.23	−1.36	5.13
1969	5.01	608.66	5.01	0.72	0.060705	0.75	616.95	−8.29	−1.36	5.07
1970	4.61	613.27	4.61	0.73	0.061202	0.77	621.98	−8.71	−1.42	5.03
1971	4.63	617.90	4.63	0.75	0.061696	0.79	626.98	−9.08	−1.47	5.00
1972	4.53	622.43	4.53	0.77	0.062185	0.81	631.93	−9.50	−1.53	4.95
1973	4.59	627.02	4.59	0.79	0.062671	0.83	636.84	−9.82	−1.57	4.91
1974	4.20	631.22	4.20	0.80	0.063153	0.85	641.72	−10.50	−1.66	4.88
1975	4.21	635.43	4.21	0.82	0.063633	0.87	646.57	−11.14	−1.75	4.85
1976	4.07	639.50	4.07	0.84	0.064109	0.88	651.39	−11.89	−1.86	4.82
1977	4.46	643.96	4.46	0.85	0.064583	0.90	656.18	−12.22	−1.90	4.79
1978	4.65	648.61	4.65	0.87	0.065055	0.92	660.96	−12.35	−1.90	4.78
1979	4.67	653.28	4.67	0.89	0.065525	0.94	665.71	−12.43	−1.90	4.75
1980	4.66	657.94	4.66	0.91	0.065993	0.96	670.44	−12.50	−1.90	4.73
1981	4.87	662.81	4.87	0.93	0.066459	0.98	675.16	−12.35	−1.86	4.72
1982	5.19	668.00	5.19	0.95	0.066924	1.00	679.86	−11.86	−1.78	4.70
1983	5.01	673.01	5.01	0.97	0.067389	1.02	684.56	−11.55	−1.72	4.70
1984	5.56	678.57	5.56	0.99	0.067853	1.03	689.26	−10.69	−1.58	4.70
1985	6.43	685.00	6.43	1.02	0.068316	1.05	693.94	−8.94	−1.31	4.68
1986	6.54	691.54	6.54	1.04	0.068780	1.07	698.64	−7.10	−1.03	4.70
1987	7.11	698.65	7.11	1.07	0.069244	1.09	703.33	−4.68	−0.67	4.69
1988	6.62	705.27	6.62	1.10	0.069708	1.11	708.02	−2.75	−0.39	4.69
1989	6.29	711.56	6.29	1.12	0.070174	1.13	712.74	−1.18	−0.17	4.72
1990	6.62	718.18	6.62	1.15	0.070640	1.15	717.45	0.73	0.10	4.71
1991	5.92	724.10	5.92	1.17	0.071109	1.16	722.19	1.91	0.26	4.74
1992	7.09	731.19	7.09	1.20	0.071579	1.18	726.95	4.24	0.58	4.76
1993	7.20	738.39	7.20	1.23	0.072052	1.20	731.73	6.66	0.90	4.78
1994	6.86	745.25	6.86	1.26	0.072527	1.22	736.54	8.71	1.17	4.81
1995	6.82	752.07	6.82	1.28	0.073006	1.24	741.38	10.69	1.42	4.84
1996	6.34	758.41	6.34	1.31	0.073488	1.26	746.26	12.15	1.60	4.88
1997	6.00	764.41	6.00	1.33	0.073974	1.28	751.17	13.24	1.73	4.91
1998	5.90	770.31	5.90	1.35	0.074465	1.30	756.14	14.17	1.84	4.97
1999	5.22	775.53	5.22	1.38	0.074961	1.32	761.16	14.37	1.85	5.02
2000	5.35	780.88	5.35	1.40	0.075462	1.34	766.22	14.66	1.88	5.06
2001	4.95	785.83	4.95	1.42	0.075969	1.36	771.35	14.48	1.84	5.13
2002	5.14	790.97	5.14	1.44	0.076483	1.38	776.55	14.42	1.82	5.20
2003	5.80	796.77	5.80	1.46	0.077004	1.40	781.82	14.95	1.88	5.27
2004	5.58	802.35	5.58	1.48	0.077533	1.42	787.17	15.18	1.89	5.35
2005	5.20	807.55	5.20	1.50	0.078070	1.44	792.61	14.94	1.85	5.44
2006	5.20	812.75	5.20	1.52	0.078617	1.46	798.14	14.61	1.80	5.53
2007	4.80	817.55	4.80	1.54	0.079173	1.49	803.76	13.79	1.69	5.62
2008	4.70	822.25	4.70	1.56	0.079741	1.51	809.51	12.74	1.55	5.75
2009	5.00	827.25	5.00	1.58	0.080320	1.53	815.36	11.89	1.44	5.85
2010	5.02	832.27	5.02	1.60	0.080912	1.56	821.35	10.92	1.31	5.99

In our case, we have

33 $${\text{S}}_{\text{t + 1}} - {\text{S}}_{\text{t}} = a{\text{S}}_{\text{t}} - bS_{\text{t}}^{2} + c{\text{S}}_{\text{t}}^{3}$$

whose solution is

34 $${\text{S}}_{\text{t + 1}} = {\text{S}}_{\text{t}} + (a{\text{S}}_{\text{t}} - b{\text{S}}_{\text{t}}^{2} + c{\text{S}}_{\text{t}}^{3}$$

Hence

35 $${\text{S}}_{\text{t + 1}} = \left( {1 + a} \right){\text{ S}}_{\text{t}} {-}b{\text{S}}_{\text{t}}^{2} + c{\text{S}}_{\text{t}}^{3}$$

or else

36 $${\text{S}}_{\text{t + 1}} = {\text{k}}_{1} {\text{S}}_{\text{t}} - {\text{ k}}_{ 2} {\text{S}}_{\text{t}}^{2} + {\text{ k}}_{ 3} {\text{S}}_{\text{t}}^{3}$$

Also in this case the model was estimated by iteration and we used the procedure which was suggested from Priesmayer. The calculation procedure was programmed so as to be interrupted at the parameter values maximizing the fit to observed data. The model showing the highest fit to observed data was the following one:

**Table:** 

Suicides 1875–2010	Obs.	*k*_1_	*k*_2_	*k*_3_	x_t_	R^2^	*M*	SD
Total	136	1.069	−1.727	11.949	0.0030	0.998	0.042	0.025

$${\text{S}}_{\text{t + 1}} = 1,069{\text{S}}_{\text{t}} - 1,727{\text{S}}_{\text{t}}^{2} + 11, \, 949 \, S_{t}^{3} \quad{\text{with}}$$

$${\text{S}}_{\text{t1}} \left( {\text{initial condition}} \right) \, = \, 0,0030\quad {\text{k}}_{ 1} = 1 + a = 1 + 0,0689\quad {\text{k}}_{ 2} = b = 1,727\quad {\text{k}}_{ 3} = c = 11,949$$

Consequently, the following parameters was estimated for the differential model $S_{t + 1} - S_{t} = aS_{t} - bS_{t}^{2} - cS_{t}^{3}$ of which the used model (35)–(36) is the solution:

$${\text{S}}_{\text{t + 1}} - {\text{S}}_{\text{t}} = \, 0,069{\text{S}}_{\text{t}} {-} \, 1,727{\text{ S}}_{\text{t}}^{ 2} + \, 11, \, 949{\text{ S}}_{\text{t}}^{ 3}$$

Now the fit is better than the previous one (R^2^ = 0.998). The efficiency of the model results evident when one examine observed and fitted data and the percent differences in the error (Table 3 in Appendix 2; Fig. 7c, d). Percent differences in the error gradually decrease, and it is smaller than the previous one even in the 80 and 90 s; when we computed R^2^ between observed and fitted data too, it accounted 99.8 % of variance (the same value of R^2^ that was computed by the *z* variables). The model led to estimate fitted suicide growth rates which represent the best fit to actual suicide growth rates (R^2^ is 0.45, but it is the highest value compared to R^2^ values that subsequently we computed by differential linear and exponential models).

In conclusion, a model which is structured in a logistic way seems to represent adequately the underlying physical mechanism of derivatives that generates suicide population data. Our analysis tested the excludability of using models structuring population change process by implying a constant growth rate (linear model and exponential model) too.

As it is known, if data is distributed in an exponential way as their growth process is a malthusian one (population grows at a increasing rate in a such way that percent differences between two following observations is constant in time), the exponential model can be estimated by linearizing data by computing their natural logarithms. Indeed, logarithmic scale allows to show a linear trend in the variable growth process. The slope *a* of the “best” linear model is the estimate of the constant of proportionality *a* (or *time constant*) of the Malthusian grow model.

**Fig. 8:**
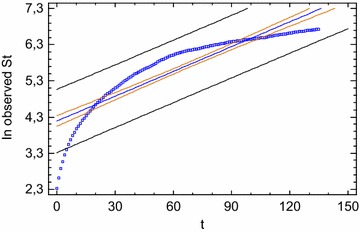
Model for in observed St

**Fig. 9:**
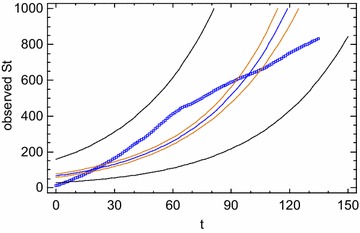
Exponential model for no logarithm scale observed data St

This being stated, we transformed our data in logarithms. The linear model that represented the best fit to the In-data had In-intercept 4.175 and slope 0.023. The model estimated a suicide growth constant or constant of proportionality of 2.3 % every years and explained 80 % of variance (R^2^ = 0.80):

$${\text{In S}}_{\text{t + 1}} = 4.175 + \, 0.023{\text{t}}$$

and computing antilogarithms

$${\text{S}}_{\text{t + 1}} = 65.039 \times {\text{ e}}^{{0.023{\text{t}}}}$$

that is,

$${\text{S}}_{\text{t + 1}} = \, 65.039 \times \left( {1.023^{\text{t}} } \right)$$

where 1.023 is natural antilogarithm of 0.023 and is equivalent to e^0.023^. Therefore, as the plot of data measured on logarithmic scale shows, the exponential model is not the best descriptor and predictor of the data (Figs. 8, 9):

The linear model

$${\text{S}}_{\text{t + 1}} = - 10.127 \, + 6.4{\text{t}}$$

does better than exponential model, explaining even 99 % of variance (with a constant suicide growth rate of about 6.5 suicide each year). This appears to conflict with what we assumed. However, as it is known, even though many model may fit the measured data (for example, a hyperbola or a parabola might fit measured data as well as an exponential curve), if the underlying physical mechanism that generates the data in not related to the model used, in this condition “the extrapolation of a curve is likely to be in error” (Kaplan and Glass 1995, p 157). In our case, since derivative or rather suicide growth rate mechanism seems not be related to the linear model because it is no a constant growth mechanism, the extrapolation of a linear curve is likely to be in error, independent of its goodness of fit. In confirmation of this, we computed the fit among actual suicide growth rates and suicide growth rates which was estimated by differential linear model as well as by differential exponential model, and R^2^ was near zero in both cases.

Finally, our model was applied to suicide data by sex too. Suicide data of other social categories (i.e. by age) was too short time series in order to test adaptation hypothesis implying the use of long time series beginning the industrialization process. The model explained the 99.9 % of variance for Male suicide population data (MS_t_, M = 676.98, SD = 388.68) and 99.6 % of variance for Female suicide population (FS_t_, M = 212.73, SD = 133.89). Following is the best estimate of parameter values and initial conditions:

**Table:** 

	Obs.	*k*_1_	*k*_2_	*k*_3_	x_t_	R^2^	*M*	SD
*M suicides 1875*–*2010*								
Total	136	1.069	1.116	5	0.0055	0.999	0.0678	0.0386
*Suicides 1875*–*2010*								
Total	136	1.057	2.2	22.8	0.0016	0.996	0.0214	0.0136

**Fig. 10:**
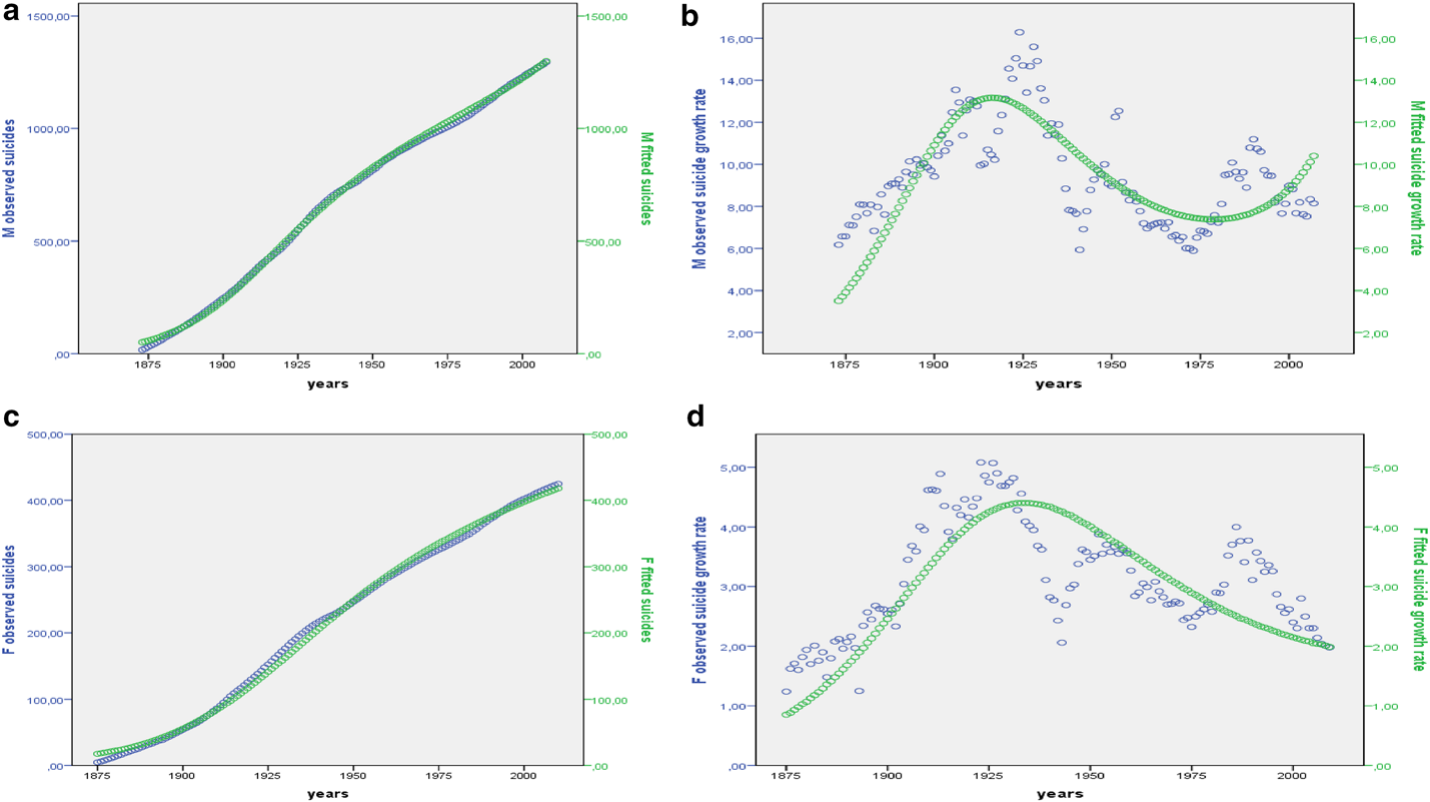
**a**, **b** Observed and fitted male and female suicide population data—MS_t_ and FS_t_; **b**, **c** Observed and fitted male and female suicide growth rates (or rather derivatives)—Ms_t_ and Fs_t_

The same R^2^ values were obtained by computing the fit among actual and fitted data too. The results are showed in Tables 4 and 5 in Appendix 2 and Fig. 10a–d.

## Conclusions

In this article we assumed that social processes result as the consequences of social structures which change in time, in the framework of a connessionist, complex, anti-reductionist conception of the micro–macro relationship. The value of this assumption transferred to suicide process can be evaluated insofar as there is a correspondence (or adequate isomorphism) between theory and formalism descriptive.

From this perspective, the findings of suicide growth rate formalization by a logistic mechanism seem to lead us to credit the adaptation hypothesis, by confirming for Italy a restrained suicide growth process. Although the analysis should be repeated also in other contexts, this is a first step to support Halbwach’s Theory. We can think that in modern society, and therefore in modern Italian society too, this process is activated. This does not mean denying the impact of Durkheim’s hypothesis, demonstrated by extensive research on the matter. The Durkheimian theory still holds. But, assuming that suicide is the most tangible sign of modernity, it means admit that the intensity with which the *liquidity* of social ties work in our lives can be now changed and Durkheim’s Theory has to be integrate with Halbwachs’ Theory. Many people *adapt* and, we repeat, this process may to be encouraged from material prosperity and materialistic cultural models shifting social interest from traditional values (working and family) on consumption. This interpretative direction seems to be supported by findings of *Bayesian Change point Analysis* too, insofar it showed that after the *economic boom* Italian suicide growth rates decreased in a *significant* way, while industrial development increased and as the individualization process intensified (marriage rates were more and more low and, beginning the 70 s, divorce and separation rates more and more high^18^). Increases in economic prosperity and consumption styles seemed to be a deterring factor on suicide, and, therefore, the fact that in Italy suicide growth rates, even though in a restrained way compared to the past growth processes, before World War II, increased just upon the onset of financial crisis in 1980s and 1990s and suicides for economic reason increased in 2008 (although they were not able to vary suicide rates in a significant way) was relevant for us. Indeed, our interpretative framework led us to expect that lack of material prosperity and an unsatisfied consumption-oriented mentality may impact on suicide decision-making processes.

In conclusion, assuming a long period perspective, a framework seems to emerge from modern Italian society. *In general* the “normality” seems to be more and more to not commit suicide due to weakening to social ties insofar they are offset by benefits of industrial progress. On the one hand, we could look at suicides as an increasingly anachronistic residue of the pre-modern past, the expression of a sensitivity to social isolation more and more decaying in a modernity which “selects the most adapted”, that is, the one able to absorb in a normal everyday life social fragmentation that characterizes modern society. However, on the other hand, there seems to be a state to which man does not adapt: poverty.

This being stated, we believe that the adaptation process to weakening to social ties finds an interesting correspondence in the baumanian analysis of modern society.

In this regard, from Bauman to Beck, contemporary sociological thought underlining an ever more *fragmentary* and *uncertain sociality*, an increasingly liquid network of social ties, characterized by a life condition transforming in a matter of taste and ever free negotiation what previously was a matter of responsibility and moral obligation, where there is no longer “history” but a collation of episodes, a culture of the ephemeral, the temporary and the uncertain. Having unstructured all the a priori traditional elements of social existence (State, Church, Family, School) and the possibility of constructing identities tied to these structures, today’s rule is what Beck calls an increasingly radical *Institutionalization of individualization process* (Beck and Beck 1994), a culture of the ephemeral which extends the materialistic mentality and the logic of consumption to social relations. However that *fluid identity* which Bauman, for example, sees as post-modern man’s condition, and which sums up the activation of a self formation process as an endless game, an ever new game, always open to new solutions without any commitment nor memory, never firmly and definitively established but open to ceaseless construction and reconstruction activity, stands out as the ultimate expression of adaptation actuated by men and women of contemporary society in response to the uncertainty of individualistic freedom. Bauman clearly says that, today, this never ending process of identity construction is increasingly being defined as a resource, as the most suitable response to a world where the art of loosing memories is increasingly the only condition to preserve well-being (Bauman 1997). This is a change of unsuspected social relevance, depriving of sense and reason those conceptual constructs that were until recently considered traditional oppositions of meaning. There are no longer winners nor losers in the game of freedom. Durkheim’s modern man lives suffering the tragedy of his freedom. For Durkheim life in modern society tends to be individualistic and more and more dangerously alienating. The more freedom he has the more he suffers this tragedy, till he reaches desperation. Instead, his life living skills within society as in a territory where he can enter and exit at will, open to a consumer approach to social relations, to ever new possibilities and to ever new redefinition of his identity, allows post-modern man to exorcise his desperation, and to stop at the threshold without going in (Bauman 1995).

In other words, possible process of adaptation of suicidal behavior to modernization shock may be considered a singular manifestation of a general trend of Post-modernity which is at the heart of contemporary sociological reflection.

Complexity, emphasizing both complexity of the individual in its cognitive processes and complexity of social systems with their non-linear social interaction property, adds something further to this already well defined picture. From our perspective, it completes it by going to the interaction structural mechanism underlying the adaptation process. In fact, whereas in the linear framework the element of *surprise* is missing, the non linearity property makes the element of *surprise* the functioning and evolution rule of social systems. So, it enables an epilogue that Durkheim could not have suspected and under a specific profile even more pessimistic. We must, in fact, delve within the deeper sense of adaptation. In this case, what does adapting really mean? To what kind of personality does the ability to adapt to individualistic freedom refer to? In conclusion, we can derive two disarming implications. The first is that even if we can show that suicides regress at a certain point in time after industrial development because of adaptation we can not however ‘shout victory’. For, in fact, man has become more and more ‘selfish’, indifferent, and cynical. The second implication concerns the problem of prevention. The prevention problem obsessing Durkheim finds paradoxically, to a certain extent, a solution, and finds it precisely in suicide’s prime cause, in the individualization process itself and in its unexpected, surprising manifestations.

## Appendix 1

See Table 1.

## Appendix 2

See Tables 2, 3, 4 and 5.

**Table 3:** Observed and fitted data by the model S_t+1_ = 1.069 × 0.0030–1.727 × 0.0030^2^ + 11.949 × 0.0030^3^

a	b (1)	b (2)	c	d	e	f	g (1)	g (2)	h
Years	Observed suicide growth rates S_t_	Observed S_t_	Z obs. S_t_ (**b** **2**)	Predicted S_t_	Z predic S_t_	Fitted S_t_	Residues **b(2)** − **f** from 1875	% Residues (**f**/**b(2**)) × 100	Fitted derivative or suicide growth rates S_t+1_ − S_t_
R^2^ = 0.998									
1873	1.39								
1874	1.87	3.26							
1875	1.28	4.54	−1.55	0.001600	−1.46	17.37	−12.83	−282.62	
1876	1.24	5.78	−1.54	0.001686	−1.45	18.22	−12.44	−215.16	0.85
1877	1.62	7.40	−1.53	0.001776	−1.45	19.10	−11.70	−158.16	0.88
1878	1.71	9.11	−1.52	0.001870	−1.44	20.03	−10.92	−119.92	0.93
1879	1.60	10.71	−1.51	0.001969	−1.43	21.01	−10.30	−96.19	0.98
1880	1.82	12.53	−1.49	0.002073	−1.42	22.04	−9.51	−75.88	1.03
1881	1.94	14.47	−1.48	0.002182	−1.42	23.11	−8.64	−59.72	1.07
1882	1.70	16.17	−1.47	0.002296	−1.41	24.24	−8.07	−49.89	1.13
1883	2.01	18.18	−1.45	0.002416	−1.40	25.42	−7.24	−39.81	1.18
1884	1.76	19.94	−1.44	0.002541	−1.39	26.65	−6.71	−33.66	1.23
1885	1.90	21.84	−1.42	0.002672	−1.38	27.94	−6.10	−27.95	1.29
1886	1.48	23.32	−1.41	0.002809	−1.37	29.30	−5.98	−25.63	1.36
1887	1.80	25.12	−1.40	0.002952	−1.36	30.71	−5.59	−22.25	1.41
1888	2.08	27.20	−1.38	0.003102	−1.35	32.19	−4.99	−18.33	1.48
1889	2.12	29.32	−1.37	0.003258	−1.34	33.73	−4.41	−15.04	1.54
1890	1.96	31.28	−1.35	0.003421	−1.32	35.34	−4.06	−12.98	1.61
1891	2.07	33.35	−1.34	0.003591	−1.31	37.02	−3.67	−11.00	1.68
1892	2.16	35.51	−1.32	0.003769	−1.30	38.77	−3.26	−9.17	1.75
1893	1.97	37.48	−1.31	0.003953	−1.29	40.59	−3.11	−8.30	1.82
1894	1.25	38.73	−1.30	0.004146	−1.27	42.49	−3.76	−9.71	1.90
1895	2.34	41.07	−1.28	0.004346	−1.26	44.46	−3.39	−8.26	1.97
1896	2.57	43.64	−1.26	0.004554	−1.24	46.52	−2.88	−6.59	2.06
1897	2.45	46.09	−1.24	0.004770	−1.23	48.65	−2.56	−5.55	2.13
1898	2.68	48.77	−1.22	0.004994	−1.21	50.86	−2.09	−4.29	2.21
1899	2.63	51.40	−1.20	0.005227	−1.19	53.16	−1.76	−3.42	2.30
1900	2.62	54.02	−1.18	0.005468	−1.17	55.54	−1.52	−2.81	2.38
1901	2.55	56.57	−1.17	0.005718	−1.16	58.00	−1.43	−2.53	2.46
1902	2.61	59.18	−1.15	0.005976	−1.14	60.55	−1.37	−2.31	2.55
1903	2.33	61.51	−1.13	0.006243	−1.12	63.18	−1.67	−2.72	2.63
1904	2.71	64.22	−1.11	0.006519	−1.10	65.90	−1.68	−2.62	2.72
1905	3.04	67.26	−1.09	0.006803	−1.08	68.71	−1.45	−2.15	2.81
1906	3.45	70.71	−1.06	0.007096	−1.05	71.60	−0.89	−1.26	2.89
1907	3.68	74.39	−1.03	0.007398	−1.03	74.58	−0.19	−0.25	2.98
1908	3.59	77.98	−1.01	0.007708	−1.01	77.64	0.34	0.44	3.06
1909	4.01	81.99	−0.98	0.008027	−0.99	80.79	1.20	1.47	3.15
1910	3.95	85.94	−0.95	0.008355	−0.96	84.02	1.92	2.23	3.23
1911	4.62	90.56	−0.91	0.008691	−0.94	87.34	3.22	3.56	3.32
1912	4.63	95.19	−0.88	0.009035	−0.91	90.73	4.46	4.68	3.39
1913	4.61	99.80	−0.84	0.009387	−0.89	94.21	5.59	5.60	3.48
1914	4.89	104.69	−0.81	0.009748	−0.86	97.76	6.93	6.62	3.55
1915	4.35	109.04	−0.77	0.010115	−0.83	101.39	7.65	7.02	3.63
1916	3.92	112.96	−0.74	0.010490	−0.80	105.09	7.87	6.97	3.70
1917	3.80	116.76	−0.72	0.010872	−0.78	108.86	7.90	6.77	3.77
1918	4.32	121.08	−0.68	0.011261	−0.75	112.70	8.38	6.92	3.84
1919	4.20	125.28	−0.65	0.011657	−0.72	116.60	8.68	6.93	3.90
1920	4.46	129.74	−0.62	0.012058	−0.69	120.56	9.18	7.08	3.96
1921	4.16	133.90	−0.59	0.012466	−0.66	124.58	9.32	6.96	4.02
1922	4.34	138.24	−0.56	0.012879	−0.63	128.65	9.59	6.93	4.07
1923	4.48	142.72	−0.52	0.013297	−0.60	132.78	9.94	6.97	4.13
1924	5.08	147.80	−0.48	0.013719	−0.57	136.95	10.85	7.34	4.17
1925	4.86	152.66	−0.45	0.014146	−0.53	141.16	11.50	7.53	4.21
1926	4.75	157.41	−0.41	0.014577	−0.50	145.41	12.00	7.63	4.25
1927	5.07	162.48	−0.37	0.015011	−0.47	149.69	12.79	7.87	4.28
1928	4.90	167.38	−0.34	0.015448	−0.44	154.00	13.38	7.99	4.31
1929	4.69	172.07	−0.30	0.015887	−0.41	158.34	13.73	7.98	4.34
1930	4.69	176.76	−0.27	0.016329	−0.37	162.70	14.06	7.96	4.36
1931	4.75	181.51	−0.23	0.016772	−0.34	167.07	14.44	7.96	4.37
1932	4.82	186.33	−0.20	0.017217	−0.31	171.46	14.87	7.98	4.39
1933	4.28	190.61	−0.16	0.017663	−0.28	175.86	14.75	7.74	4.40
1934	4.56	195.17	−0.13	0.018109	−0.24	180.26	14.91	7.64	4.40
1935	4.09	199.26	−0.10	0.018555	−0.21	184.66	14.60	7.33	4.40
1936	4.02	203.28	−0.07	0.019001	−0.18	189.06	14.22	7.00	4.40
1937	3.95	207.23	−0.04	0.019446	−0.14	193.45	13.78	6.65	4.39
1938	3.68	210.91	−0.01	0.019890	−0.11	197.83	13.08	6.20	4.38
1939	3.62	214.53	0.01	0.020333	−0.08	202.20	12.33	5.75	4.37
1940	3.11	217.64	0.04	0.020774	−0.05	206.55	11.09	5.09	4.35
1941	2.82	220.46	0.06	0.021213	−0.01	210.89	9.57	4.34	4.34
1942	2.77	223.23	0.08	0.021650	0.02	215.20	8.03	3.60	4.31
1943	2.43	225.66	0.10	0.022084	0.05	219.48	6.18	2.74	4.28
1944	2.06	227.72	0.11	0.022516	0.08	223.74	3.98	1.75	4.26
1945	2.69	230.41	0.13	0.022944	0.11	227.96	2.45	1.06	4.22
1946	2.97	233.38	0.15	0.023369	0.15	232.16	1.22	0.52	4.20
1947	3.03	236.41	0.18	0.023790	0.18	236.32	0.09	0.04	4.16
1948	3.38	239.79	0.20	0.024208	0.21	240.44	−0.65	−0.27	4.12
1949	3.62	243.41	0.23	0.024622	0.24	244.52	−1.11	−0.46	4.08
1950	3.58	246.99	0.26	0.025032	0.27	248.57	−1.58	−0.64	4.05
1951	3.45	250.44	0.28	0.025438	0.30	252.58	−2.14	−0.85	4.01
1952	3.51	253.95	0.31	0.025840	0.33	256.54	−2.59	−1.02	3.96
1953	3.88	257.83	0.34	0.026237	0.36	260.46	−2.63	−1.02	3.92
1954	3.55	261.38	0.36	0.026630	0.39	264.33	−2.95	−1.13	3.87
1955	3.70	265.08	0.39	0.027019	0.41	268.17	−3.09	−1.16	3.84
1956	3.58	268.66	0.42	0.027402	0.44	271.95	−3.29	−1.23	3.78
1957	3.67	272.33	0.44	0.027781	0.47	275.69	−3.36	−1.23	3.74
1958	3.55	275.88	0.47	0.028156	0.50	279.39	−3.51	−1.27	3.70
1959	3.61	279.49	0.50	0.028526	0.53	283.04	−3.55	−1.27	3.65
1960	3.56	283.05	0.52	0.028891	0.55	286.64	−3.59	−1.27	3.60
1961	3.27	286.32	0.55	0.029251	0.58	290.19	−3.87	−1.35	3.55
1962	2.84	289.16	0.57	0.029606	0.60	293.70	−4.54	−1.57	3.51
1963	2.90	292.06	0.59	0.029957	0.63	297.16	−5.10	−1.75	3.46
1964	3.05	295.11	0.61	0.030304	0.66	300.58	−5.47	−1.85	3.42
1965	2.99	298.10	0.64	0.030645	0.68	303.95	−5.85	−1.96	3.37
1966	2.77	300.87	0.66	0.030982	0.71	307.27	−6.40	−2.13	3.32
1967	3.08	303.95	0.68	0.031314	0.73	310.55	−6.60	−2.17	3.28
1968	2.92	306.87	0.70	0.031642	0.75	313.78	−6.91	−2.25	3.23
1969	2.82	309.69	0.72	0.031965	0.78	316.97	−7.28	−2.35	3.19
1970	2.70	312.39	0.74	0.032284	0.80	320.12	−7.73	−2.47	3.15
1971	2.71	315.10	0.76	0.032598	0.83	323.22	−8.12	−2.58	3.10
1972	2.75	317.85	0.78	0.032908	0.85	326.28	−8.43	−2.65	3.06
1973	2.72	320.57	0.81	0.033214	0.87	329.30	−8.73	−2.72	3.02
1974	2.44	323.01	0.82	0.033516	0.89	332.27	−9.26	−2.87	2.97
1975	2.48	325.49	0.84	0.033813	0.91	335.21	−9.72	−2.99	2.94
1976	2.32	327.81	0.86	0.034107	0.94	338.10	−10.29	−3.14	2.89
1977	2.50	330.31	0.88	0.034396	0.96	340.96	−10.65	−3.22	2.86
1978	2.56	332.87	0.90	0.034682	0.98	343.78	−10.91	−3.28	2.82
1979	2.62	335.49	0.92	0.034964	1.00	346.56	−11.07	−3.30	2.78
1980	2.70	338.19	0.94	0.035242	1.02	349.30	−11.11	−3.29	2.74
1981	2.58	340.77	0.96	0.035516	1.04	352.01	−11.24	−3.30	2.71
1982	2.90	343.67	0.98	0.035787	1.06	354.68	−11.01	−3.20	2.67
1983	2.89	346.56	1.00	0.036054	1.08	357.32	−10.76	−3.10	2.64
1984	3.03	349.59	1.02	0.036318	1.10	359.92	−10.33	−2.96	2.60
1985	3.52	353.11	1.05	0.036579	1.12	362.49	−9.38	−2.66	2.57
1986	3.70	356.81	1.08	0.036836	1.14	365.03	−8.22	−2.30	2.54
1987	4.00	360.81	1.11	0.037090	1.16	367.54	−6.73	−1.86	2.51
1988	3.76	364.57	1.13	0.037341	1.17	370.01	−5.44	−1.49	2.47
1989	3.41	367.98	1.16	0.037589	1.19	372.46	−4.48	−1.22	2.45
1990	3.77	371.75	1.19	0.037834	1.21	374.88	−3.13	−0.84	2.42
1991	3.11	374.86	1.21	0.038076	1.23	377.27	−2.41	−0.64	2.39
1992	3.57	378.43	1.24	0.038316	1.25	379.63	−1.20	−0.32	2.36
1993	3.43	381.86	1.26	0.038552	1.26	381.96	−0.10	−0.03	2.33
1994	3.25	385.11	1.29	0.038786	1.28	384.27	0.84	0.22	2.31
1995	3.36	388.47	1.31	0.039018	1.30	386.56	1.91	0.49	2.29
1996	3.26	391.73	1.34	0.039247	1.32	388.82	2.91	0.74	2.26
1997	2.87	394.60	1.36	0.039474	1.33	391.06	3.54	0.90	2.24
1998	2.66	397.26	1.38	0.039698	1.35	393.27	3.99	1.00	2.21
1999	2.56	399.82	1.40	0.039920	1.36	395.46	4.36	1.09	2.19
2000	2.62	402.44	1.42	0.040140	1.38	397.63	4.81	1.19	2.17
2001	2.40	404.84	1.43	0.040358	1.40	399.78	5.06	1.25	2.15
2002	2.30	407.14	1.45	0.040574	1.41	401.91	5.23	1.28	2.13
2003	2.80	409.94	1.47	0.040788	1.43	404.02	5.92	1.44	2.11
2004	2.50	412.44	1.49	0.041000	1.44	406.11	6.33	1.53	2.09
2005	2.30	414.74	1.51	0.041210	1.46	408.19	6.55	1.58	2.08
2006	2.30	417.04	1.52	0.041418	1.48	410.24	6.80	1.63	2.05
2007	2.14	419.18	1.54	0.041625	1.49	412.28	6.90	1.64	2.04
2008	2.04	421.22	1.56	0.041830	1.51	414.31	6.91	1.64	2.03
2009	2.01	423.23	1.57	0.042034	1.52	416.32	6.91	1.63	2.01
2010	1.98	425.21	1.59	0.042236	1.54	418.31	6.90	1.62	1.99

**Table 4:** Observed and fitted male suicide data by model S_t+1_ = 1.069 × 0.0055–1.116 × 0.0055^2^ + 5 × 0.0055^3^

a	b(1)	b(2)	c	d	e	f	g (1)	g (2)	h
Years	M Observed suicide growth rates s_t_	M Observed S_t_	zM Obs.S_t_ (**b** and **f**)	M Predicted S_t_	zM Predic S_t_	M Fitted St	Residues **b(2)** − **f** from 1875	% Residues (**f**/**b(2**)) × 100	M Fitted derivative or suicide growth rates S_t+1_ − S_t_
R^2^ = 0.999									
1873	5.80								
1874	5.57	11.37							
1875	5.43	16.80	−1.70	0.005500	−1.61	49.65	−32.85	−195.56	
1876	6.18	22.98	−1.68	0.005847	−1.61	53.14	−30.16	−131.26	3.49
1877	6.58	29.56	−1.67	0.006213	−1.60	56.83	−27.27	−92.26	3.69
1878	6.58	36.14	−1.65	0.006600	−1.59	60.73	−24.59	−68.03	3.90
1879	7.12	43.26	−1.63	0.007008	−1.57	64.84	−21.58	−49.88	4.11
1880	7.10	50.36	−1.61	0.007438	−1.56	69.17	−18.81	−37.36	4.33
1881	7.51	57.87	−1.59	0.007892	−1.55	73.74	−15.87	−27.42	4.57
1882	8.10	65.97	−1.57	0.008369	−1.54	78.55	−12.58	−19.07	4.81
1883	8.09	74.06	−1.55	0.008872	−1.53	83.60	−9.54	−12.89	5.05
1884	7.68	81.74	−1.53	0.009399	−1.51	88.92	−7.18	−8.78	5.32
1885	8.09	89.83	−1.51	0.009954	−1.50	94.50	−4.67	−5.20	5.58
1886	6.84	96.67	−1.49	0.010535	−1.48	100.35	−3.68	−3.81	5.85
1887	7.98	104.65	−1.47	0.011144	−1.47	106.48	−1.83	−1.75	6.13
1888	8.58	113.23	−1.45	0.011781	−1.45	112.90	0.33	0.29	6.42
1889	7.62	120.85	−1.43	0.012447	−1.43	119.61	1.24	1.03	6.71
1890	8.97	129.82	−1.41	0.013143	−1.42	126.61	3.21	2.47	7.00
1891	9.08	138.90	−1.38	0.013868	−1.40	133.92	4.98	3.59	7.31
1892	9.09	147.99	−1.36	0.014624	−1.38	141.52	6.47	4.37	7.60
1893	9.29	157.28	−1.34	0.015410	−1.36	149.44	7.84	4.99	7.92
1894	8.90	166.18	−1.31	0.016226	−1.34	157.66	8.52	5.13	8.22
1895	9.65	175.83	−1.29	0.017073	−1.31	166.19	9.64	5.48	8.53
1896	10.15	185.98	−1.26	0.017951	−1.29	175.03	10.95	5.89	8.84
1897	9.52	195.50	−1.24	0.018859	−1.27	184.17	11.33	5.80	9.14
1898	10.24	205.74	−1.21	0.019797	−1.24	193.61	12.13	5.89	9.44
1899	9.96	215.70	−1.19	0.020764	−1.22	203.36	12.34	5.72	9.75
1900	10.03	225.73	−1.16	0.021760	−1.19	213.39	12.34	5.47	10.03
1901	9.86	235.59	−1.14	0.022785	−1.17	223.70	11.89	5.05	10.31
1902	9.72	245.31	−1.11	0.023837	−1.14	234.30	11.01	4.49	10.60
1903	9.43	254.74	−1.09	0.024915	−1.11	245.15	9.59	3.76	10.85
1904	10.42	265.16	−1.06	0.026019	−1.08	256.27	8.89	3.35	11.12
1905	11.37	276.53	−1.03	0.027147	−1.05	267.63	8.90	3.22	11.36
1906	10.65	287.18	−1.00	0.028298	−1.02	279.21	7.97	2.77	11.58
1907	11.00	298.18	−0.97	0.029470	−0.99	291.02	7.16	2.40	11.81
1908	12.48	310.66	−0.94	0.030662	−0.96	303.02	7.64	2.46	12.00
1909	13.55	324.21	−0.91	0.031872	−0.93	315.21	9.00	2.78	12.19
1910	12.95	337.16	−0.87	0.033100	−0.90	327.57	9.59	2.84	12.36
1911	11.38	348.54	−0.85	0.034342	−0.87	340.08	8.46	2.43	12.51
1912	12.57	361.11	−0.81	0.035598	−0.83	352.73	8.38	2.32	12.65
1913	13.08	374.19	−0.78	0.036866	−0.80	365.49	8.70	2.32	12.76
1914	13.00	387.19	−0.75	0.038143	−0.77	378.36	8.83	2.28	12.87
1915	12.78	399.97	−0.71	0.039429	−0.73	391.30	8.67	2.17	12.94
1916	9.96	409.93	−0.69	0.040721	−0.70	404.31	5.62	1.37	13.01
1917	10.03	419.96	−0.66	0.042018	−0.67	417.37	2.59	0.62	13.06
1918	10.70	430.66	−0.63	0.043318	−0.63	430.46	0.20	0.05	13.09
1919	10.47	441.13	−0.61	0.044619	−0.60	443.56	−2.43	−0.55	13.10
1920	10.23	451.36	−0.58	0.045920	−0.57	456.66	−5.30	−1.17	13.10
1921	11.59	462.95	−0.55	0.047220	−0.53	469.75	−6.80	−1.47	13.09
1922	12.35	475.30	−0.52	0.048516	−0.50	482.80	−7.50	−1.58	13.05
1923	13.12	488.42	−0.49	0.049808	−0.47	495.81	−7.39	−1.51	13.01
1924	14.56	502.98	−0.45	0.051094	−0.43	508.76	−5.78	−1.15	12.95
1925	14.08	517.06	−0.41	0.052373	−0.40	521.63	−4.57	−0.88	12.87
1926	15.05	532.11	−0.37	0.053643	−0.37	534.43	−2.32	−0.44	12.80
1927	16.29	548.40	−0.33	0.054905	−0.33	547.14	1.26	0.23	12.71
1928	14.71	563.11	−0.29	0.056157	−0.30	559.74	3.37	0.60	12.60
1929	13.42	576.53	−0.26	0.057398	−0.27	572.24	4.29	0.74	12.50
1930	14.67	591.20	−0.22	0.058627	−0.24	584.61	6.59	1.11	12.37
1931	15.60	606.80	−0.18	0.059844	−0.21	596.87	9.93	1.64	12.26
1932	14.92	621.72	−0.14	0.061048	−0.17	608.99	12.73	2.05	12.12
1933	13.62	635.34	−0.11	0.062239	−0.14	620.98	14.36	2.26	11.99
1934	13.05	648.39	−0.07	0.063416	−0.11	632.83	15.56	2.40	11.85
1935	11.38	659.77	−0.04	0.064579	−0.08	644.54	15.23	2.31	11.71
1936	11.95	671.72	−0.01	0.065727	−0.05	656.11	15.61	2.32	11.57
1937	11.40	683.12	0.02	0.066861	−0.02	667.52	15.60	2.28	11.41
1938	11.90	695.02	0.05	0.067980	0.00	678.79	16.23	2.34	11.27
1939	10.29	705.31	0.07	0.069084	0.03	689.91	15.40	2.18	11.12
1940	8.85	714.16	0.10	0.070173	0.06	700.87	13.29	1.86	10.96
1941	7.83	721.99	0.12	0.071247	0.09	711.69	10.30	1.43	10.82
1942	7.79	729.78	0.14	0.072306	0.12	722.36	7.42	1.02	10.67
1943	7.66	737.44	0.16	0.073351	0.14	732.88	4.56	0.62	10.52
1944	5.94	743.38	0.17	0.074381	0.17	743.25	0.13	0.02	10.37
1945	6.92	750.30	0.19	0.075397	0.20	753.47	−3.17	−0.42	10.22
1946	7.78	758.08	0.21	0.076398	0.22	763.56	−5.48	−0.72	10.09
1947	8.79	766.87	0.23	0.077385	0.25	773.50	−6.63	−0.86	9.94
1948	9.28	776.15	0.26	0.078359	0.27	783.30	−7.15	−0.92	9.80
1949	9.75	785.90	0.28	0.079319	0.30	792.97	−7.07	−0.90	9.67
1950	9.52	795.42	0.30	0.080266	0.32	802.50	−7.08	−0.89	9.53
1951	10.01	805.43	0.33	0.081200	0.35	811.91	−6.48	−0.80	9.41
1952	9.09	814.52	0.35	0.082121	0.37	821.19	−6.67	−0.82	9.28
1953	8.97	823.49	0.38	0.083030	0.39	830.34	−6.85	−0.83	9.15
1954	12.26	835.75	0.41	0.083928	0.42	839.38	−3.63	−0.43	9.04
1955	12.55	848.30	0.44	0.084814	0.44	848.30	0.00	0.00	8.92
1956	9.16	857.46	0.46	0.085689	0.46	857.11	0.35	0.04	8.81
1957	8.66	866.12	0.49	0.086553	0.49	865.81	0.31	0.04	8.70
1958	8.30	874.42	0.51	0.087406	0.51	874.41	0.01	0.00	8.60
1959	8.64	883.06	0.53	0.088250	0.53	882.90	0.16	0.02	8.49
1960	8.24	891.30	0.55	0.089085	0.55	891.30	0.00	0.00	8.40
1961	7.78	899.08	0.57	0.089910	0.57	899.61	−0.53	−0.06	8.31
1962	7.20	906.28	0.59	0.090726	0.59	907.83	−1.55	−0.17	8.22
1963	6.96	913.24	0.61	0.091534	0.61	915.97	−2.73	−0.30	8.14
1964	7.08	920.32	0.63	0.092334	0.64	924.02	−3.70	−0.40	8.05
1965	7.16	927.48	0.64	0.093127	0.66	932.00	−4.52	−0.49	7.98
1966	7.22	934.70	0.66	0.093912	0.68	939.91	−5.21	−0.56	7.91
1967	7.24	941.94	0.68	0.094691	0.70	947.75	−5.81	−0.62	7.84
1968	6.95	948.89	0.70	0.095463	0.72	955.53	−6.64	−0.70	7.78
1969	7.25	956.14	0.72	0.096229	0.74	963.25	−7.11	−0.74	7.72
1970	6.57	962.71	0.74	0.096990	0.76	970.91	−8.20	−0.85	7.66
1971	6.64	969.35	0.75	0.097746	0.78	978.52	−9.17	−0.95	7.61
1972	6.38	975.73	0.77	0.098498	0.80	986.09	−10.36	−1.06	7.57
1973	6.55	982.28	0.79	0.099245	0.81	993.61	−11.33	−1.15	7.52
1974	6.02	988.30	0.80	0.099988	0.83	1001.10	−12.80	−1.29	7.49
1975	6.01	994.31	0.82	0.100728	0.85	1008.55	−14.24	−1.43	7.45
1976	5.90	1000.21	0.83	0.101466	0.87	1015.97	−15.76	−1.58	7.42
1977	6.52	1006.73	0.85	0.102200	0.89	1023.37	−16.64	−1.65	7.40
1978	6.85	1013.58	0.87	0.102933	0.91	1030.75	−17.17	−1.69	7.38
1979	6.82	1020.40	0.88	0.103664	0.93	1038.11	−17.71	−1.74	7.36
1980	6.71	1027.11	0.90	0.104394	0.95	1045.46	−18.35	−1.79	7.35
1981	7.28	1034.39	0.92	0.105123	0.97	1052.81	−18.42	−1.78	7.35
1982	7.58	1041.97	0.94	0.105853	0.99	1060.15	−18.18	−1.74	7.34
1983	7.24	1049.21	0.96	0.106582	1.00	1067.49	−18.28	−1.74	7.34
1984	8.12	1057.33	0.98	0.107313	1.02	1074.85	−17.52	−1.66	7.36
1985	9.50	1066.83	1.00	0.108044	1.04	1082.22	−15.39	−1.44	7.37
1986	9.54	1076.37	1.03	0.108778	1.06	1089.61	−13.24	−1.23	7.39
1987	10.09	1086.46	1.05	0.109514	1.08	1097.02	−10.56	−0.97	7.41
1988	9.65	1096.11	1.08	0.110253	1.10	1104.46	−8.35	−0.76	7.44
1989	9.32	1105.43	1.10	0.110996	1.12	1111.94	−6.51	−0.59	7.48
1990	9.63	1115.06	1.13	0.111743	1.14	1119.46	−4.40	−0.39	7.52
1991	8.90	1123.96	1.15	0.112495	1.16	1127.03	−3.07	−0.27	7.57
1992	10.80	1134.76	1.18	0.113252	1.18	1134.65	0.11	0.01	7.62
1993	11.20	1145.96	1.21	0.114015	1.20	1142.34	3.62	0.32	7.69
1994	10.75	1156.71	1.23	0.114786	1.22	1150.10	6.61	0.57	7.76
1995	10.61	1167.32	1.26	0.115564	1.24	1157.93	9.39	0.80	7.83
1996	9.73	1177.05	1.29	0.116350	1.26	1165.85	11.20	0.95	7.92
1997	9.48	1186.53	1.31	0.117146	1.28	1173.87	12.66	1.07	8.02
1998	9.46	1195.99	1.34	0.117952	1.30	1181.98	14.01	1.17	8.11
1999	8.19	1204.18	1.36	0.118769	1.32	1190.21	13.97	1.16	8.23
2000	8.42	1212.60	1.38	0.119599	1.34	1198.56	14.04	1.16	8.35
2001	7.67	1220.27	1.40	0.120442	1.36	1207.05	13.22	1.08	8.49
2002	8.13	1228.40	1.42	0.121299	1.39	1215.68	12.72	1.04	8.63
2003	8.99	1237.39	1.44	0.122172	1.41	1224.47	12.92	1.04	8.79
2004	8.83	1246.22	1.46	0.123062	1.43	1233.44	12.78	1.03	8.97
2005	7.68	1253.90	1.48	0.123971	1.46	1242.59	11.31	0.90	9.15
2006	8.20	1262.10	1.51	0.124900	1.48	1251.94	10.16	0.80	9.35
2007	7.63	1269.73	1.53	0.125850	1.50	1261.51	8.22	0.65	9.57
2008	7.53	1277.26	1.54	0.126825	1.53	1271.33	5.93	0.46	9.82
2009	8.34	1285.60	1.57	0.127825	1.56	1281.40	4.20	0.33	10.07
2010	8.15	1293.75	1.59	0.128853	1.58	1291.75	2.00	0.15	10.35

**Table 5:** Observed and fitted Female suicide data by model S_t+1_ = 1.057 × 0.0016–2.2 × 0.0016^2^ + 22.8 × 0.0016^3^

a	b(1)	b(2)	c	d	e	f	g (1)	g (2)	h
Years	F Observed suicide growth rates s_t_	F Observed S_t_	zF obs.S_t_ (**b** and **f**)	F Predicted S_t_	zF predic S_t_	F Fitted	Residues **b(2)** − **f** from 1875	% Residues (**f**/**b(2**)) × 100	F Fitted derivative or suicide growth rates S_t+1_ − S_t_
R^2^ = 0.996									
1873	1.39								
1874	1.87	3.26							
1875	1.28	4.54	−1.55	0.001600	−1.46	17.37	−12.83	−282.62	
1876	1.24	5.78	−1.54	0.001686	−1.45	18.22	−12.44	−215.16	0.85
1877	1.62	7.40	−1.53	0.001776	−1.45	19.10	−11.70	−158.16	0.88
1878	1.71	9.11	−1.52	0.001870	−1.44	20.03	−10.92	−119.92	0.93
1879	1.60	10.71	−1.51	0.001969	−1.43	21.01	−10.30	−96.19	0.98
1880	1.82	12.53	−1.49	0.002073	−1.42	22.04	−9.51	−75.88	1.03
1881	1.94	14.47	−1.48	0.002182	−1.42	23.11	−8.64	−59.72	1.07
1882	1.70	16.17	−1.47	0.002296	−1.41	24.24	−8.07	−49.89	1.13
1883	2.01	18.18	−1.45	0.002416	−1.40	25.42	−7.24	−39.81	1.18
1884	1.76	19.94	−1.44	0.002541	−1.39	26.65	−6.71	−33.66	1.23
1885	1.90	21.84	−1.42	0.002672	−1.38	27.94	−6.10	−27.95	1.29
1886	1.48	23.32	−1.41	0.002809	−1.37	29.30	−5.98	−25.63	1.36
1887	1.80	25.12	−1.40	0.002952	−1.36	30.71	−5.59	−22.25	1.41
1888	2.08	27.20	−1.38	0.003102	−1.35	32.19	−4.99	−18.33	1.48
1889	2.12	29.32	−1.37	0.003258	−1.34	33.73	−4.41	−15.04	1.54
1890	1.96	31.28	−1.35	0.003421	−1.32	35.34	−4.06	−12.98	1.61
1891	2.07	33.35	−1.34	0.003591	−1.31	37.02	−3.67	−11.00	1.68
1892	2.16	35.51	−1.32	0.003769	−1.30	38.77	−3.26	−9.17	1.75
1893	1.97	37.48	−1.31	0.003953	−1.29	40.59	−3.11	−8.30	1.82
1894	1.25	38.73	−1.30	0.004146	−1.27	42.49	−3.76	−9.71	1.90
1895	2.34	41.07	−1.28	0.004346	−1.26	44.46	−3.39	−8.26	1.97
1896	2.57	43.64	−1.26	0.004554	−1.24	46.52	−2.88	−6.59	2.06
1897	2.45	46.09	−1.24	0.004770	−1.23	48.65	−2.56	−5.55	2.13
1898	2.68	48.77	−1.22	0.004994	−1.21	50.86	−2.09	−4.29	2.21
1899	2.63	51.40	−1.20	0.005227	−1.19	53.16	−1.76	−3.42	2.30
1900	2.62	54.02	−1.18	0.005468	−1.17	55.54	−1.52	−2.81	2.38
1901	2.55	56.57	−1.17	0.005718	−1.16	58.00	−1.43	−2.53	2.46
1902	2.61	59.18	−1.15	0.005976	−1.14	60.55	−1.37	−2.31	2.55
1903	2.33	61.51	−1.13	0.006243	−1.12	63.18	−1.67	−2.72	2.63
1904	2.71	64.22	−1.11	0.006519	−1.10	65.90	−1.68	−2.62	2.72
1905	3.04	67.26	−1.09	0.006803	−1.08	68.71	−1.45	−2.15	2.81
1906	3.45	70.71	−1.06	0.007096	−1.05	71.60	−0.89	−1.26	2.89
1907	3.68	74.39	−1.03	0.007398	−1.03	74.58	−0.19	−0.25	2.98
1908	3.59	77.98	−1.01	0.007708	−1.01	77.64	0.34	0.44	3.06
1909	4.01	81.99	−0.98	0.008027	−0.99	80.79	1.20	1.47	3.15
1910	3.95	85.94	−0.95	0.008355	−0.96	84.02	1.92	2.23	3.23
1911	4.62	90.56	−0.91	0.008691	−0.94	87.34	3.22	3.56	3.32
1912	4.63	95.19	−0.88	0.009035	−0.91	90.73	4.46	4.68	3.39
1913	4.61	99.80	−0.84	0.009387	−0.89	94.21	5.59	5.60	3.48
1914	4.89	104.69	−0.81	0.009748	−0.86	97.76	6.93	6.62	3.55
1915	4.35	109.04	−0.77	0.010115	−0.83	101.39	7.65	7.02	3.63
1916	3.92	112.96	−0.74	0.010490	−0.80	105.09	7.87	6.97	3.70
1917	3.80	116.76	−0.72	0.010872	−0.78	108.86	7.90	6.77	3.77
1918	4.32	121.08	−0.68	0.011261	−0.75	112.70	8.38	6.92	3.84
1919	4.20	125.28	−0.65	0.011657	−0.72	116.60	8.68	6.93	3.90
1920	4.46	129.74	−0.62	0.012058	−0.69	120.56	9.18	7.08	3.96
1921	4.16	133.90	−0.59	0.012466	−0.66	124.58	9.32	6.96	4.02
1922	4.34	138.24	−0.56	0.012879	−0.63	128.65	9.59	6.93	4.07
1923	4.48	142.72	−0.52	0.013297	−0.60	132.78	9.94	6.97	4.13
1924	5.08	147.80	−0.48	0.013719	−0.57	136.95	10.85	7.34	4.17
1925	4.86	152.66	−0.45	0.014146	−0.53	141.16	11.50	7.53	4.21
1926	4.75	157.41	−0.41	0.014577	−0.50	145.41	12.00	7.63	4.25
1927	5.07	162.48	−0.37	0.015011	−0.47	149.69	12.79	7.87	4.28
1928	4.90	167.38	−0.34	0.015448	−0.44	154.00	13.38	7.99	4.31
1929	4.69	172.07	−0.30	0.015887	−0.41	158.34	13.73	7.98	4.34
1930	4.69	176.76	−0.27	0.016329	−0.37	162.70	14.06	7.96	4.36
1931	4.75	181.51	−0.23	0.016772	−0.34	167.07	14.44	7.96	4.37
1932	4.82	186.33	−0.20	0.017217	−0.31	171.46	14.87	7.98	4.39
1933	4.28	190.61	−0.16	0.017663	−0.28	175.86	14.75	7.74	4.40
1934	4.56	195.17	−0.13	0.018109	−0.24	180.26	14.91	7.64	4.40
1935	4.09	199.26	−0.10	0.018555	−0.21	184.66	14.60	7.33	4.40
1936	4.02	203.28	−0.07	0.019001	−0.18	189.06	14.22	7.00	4.40
1937	3.95	207.23	−0.04	0.019446	−0.14	193.45	13.78	6.65	4.39
1938	3.68	210.91	−0.01	0.019890	−0.11	197.83	13.08	6.20	4.38
1939	3.62	214.53	0.01	0.020333	−0.08	202.20	12.33	5.75	4.37
1940	3.11	217.64	0.04	0.020774	−0.05	206.55	11.09	5.09	4.35
1941	2.82	220.46	0.06	0.021213	−0.01	210.89	9.57	4.34	4.34
1942	2.77	223.23	0.08	0.021650	0.02	215.20	8.03	3.60	4.31
1943	2.43	225.66	0.10	0.022084	0.05	219.48	6.18	2.74	4.28
1944	2.06	227.72	0.11	0.022516	0.08	223.74	3.98	1.75	4.26
1945	2.69	230.41	0.13	0.022944	0.11	227.96	2.45	1.06	4.22
1946	2.97	233.38	0.15	0.023369	0.15	232.16	1.22	0.52	4.20
1947	3.03	236.41	0.18	0.023790	0.18	236.32	0.09	0.04	4.16
1948	3.38	239.79	0.20	0.024208	0.21	240.44	−0.65	−0.27	4.12
1949	3.62	243.41	0.23	0.024622	0.24	244.52	−1.11	−0.46	4.08
1950	3.58	246.99	0.26	0.025032	0.27	248.57	−1.58	−0.64	4.05
1951	3.45	250.44	0.28	0.025438	0.30	252.58	−2.14	−0.85	4.01
1952	3.51	253.95	0.31	0.025840	0.33	256.54	−2.59	−1.02	3.96
1953	3.88	257.83	0.34	0.026237	0.36	260.46	−2.63	−1.02	3.92
1954	3.55	261.38	0.36	0.026630	0.39	264.33	−2.95	−1.13	3.87
1955	3.70	265.08	0.39	0.027019	0.41	268.17	−3.09	−1.16	3.84
1956	3.58	268.66	0.42	0.027402	0.44	271.95	−3.29	−1.23	3.78
1957	3.67	272.33	0.44	0.027781	0.47	275.69	−3.36	−1.23	3.74
1958	3.55	275.88	0.47	0.028156	0.50	279.39	−3.51	−1.27	3.70
1959	3.61	279.49	0.50	0.028526	0.53	283.04	−3.55	−1.27	3.65
1960	3.56	283.05	0.52	0.028891	0.55	286.64	−3.59	−1.27	3.60
1961	3.27	286.32	0.55	0.029251	0.58	290.19	−3.87	−1.35	3.55
1962	2.84	289.16	0.57	0.029606	0.60	293.70	−4.54	−1.57	3.51
1963	2.90	292.06	0.59	0.029957	0.63	297.16	−5.10	−1.75	3.46
1964	3.05	295.11	0.61	0.030304	0.66	300.58	−5.47	−1.85	3.42
1965	2.99	298.10	0.64	0.030645	0.68	303.95	−5.85	−1.96	3.37
1966	2.77	300.87	0.66	0.030982	0.71	307.27	−6.40	−2.13	3.32
1967	3.08	303.95	0.68	0.031314	0.73	310.55	−6.60	−2.17	3.28
1968	2.92	306.87	0.70	0.031642	0.75	313.78	−6.91	−2.25	3.23
1969	2.82	309.69	0.72	0.031965	0.78	316.97	−7.28	−2.35	3.19
1970	2.70	312.39	0.74	0.032284	0.80	320.12	−7.73	−2.47	3.15
1971	2.71	315.10	0.76	0.032598	0.83	323.22	−8.12	−2.58	3.10
1972	2.75	317.85	0.78	0.032908	0.85	326.28	−8.43	−2.65	3.06
1973	2.72	320.57	0.81	0.033214	0.87	329.30	−8.73	−2.72	3.02
1974	2.44	323.01	0.82	0.033516	0.89	332.27	−9.26	−2.87	2.97
1975	2.48	325.49	0.84	0.033813	0.91	335.21	−9.72	−2.99	2.94
1976	2.32	327.81	0.86	0.034107	0.94	338.10	−10.29	−3.14	2.89
1977	2.50	330.31	0.88	0.034396	0.96	340.96	−10.65	−3.22	2.86
1978	2.56	332.87	0.90	0.034682	0.98	343.78	−10.91	−3.28	2.82
1979	2.62	335.49	0.92	0.034964	1.00	346.56	−11.07	−3.30	2.78
1980	2.70	338.19	0.94	0.035242	1.02	349.30	−11.11	−3.29	2.74
1981	2.58	340.77	0.96	0.035516	1.04	352.01	−11.24	−3.30	2.71
1982	2.90	343.67	0.98	0.035787	1.06	354.68	−11.01	−3.20	2.67
1983	2.89	346.56	1.00	0.036054	1.08	357.32	−10.76	−3.10	2.64
1984	3.03	349.59	1.02	0.036318	1.10	359.92	−10.33	−2.96	2.60
1985	3.52	353.11	1.05	0.036579	1.12	362.49	−9.38	−2.66	2.57
1986	3.70	356.81	1.08	0.036836	1.14	365.03	−8.22	−2.30	2.54
1987	4.00	360.81	1.11	0.037090	1.16	367.54	−6.73	−1.86	2.51
1988	3.76	364.57	1.13	0.037341	1.17	370.01	−5.44	−1.49	2.47
1989	3.41	367.98	1.16	0.037589	1.19	372.46	−4.48	−1.22	2.45
1990	3.77	371.75	1.19	0.037834	1.21	374.88	−3.13	−0.84	2.42
1991	3.11	374.86	1.21	0.038076	1.23	377.27	−2.41	−0.64	2.39
1992	3.57	378.43	1.24	0.038316	1.25	379.63	−1.20	−0.32	2.36
1993	3.43	381.86	1.26	0.038552	1.26	381.96	−0.10	−0.03	2.33
1994	3.25	385.11	1.29	0.038786	1.28	384.27	0.84	0.22	2.31
1995	3.36	388.47	1.31	0.039018	1.30	386.56	1.91	0.49	2.29
1996	3.26	391.73	1.34	0.039247	1.32	388.82	2.91	0.74	2.26
1997	2.87	394.60	1.36	0.039474	1.33	391.06	3.54	0.90	2.24
1998	2.66	397.26	1.38	0.039698	1.35	393.27	3.99	1.00	2.21
1999	2.56	399.82	1.40	0.039920	1.36	395.46	4.36	1.09	2.19
2000	2.62	402.44	1.42	0.040140	1.38	397.63	4.81	1.19	2.17
2001	2.40	404.84	1.43	0.040358	1.40	399.78	5.06	1.25	2.15
2002	2.30	407.14	1.45	0.040574	1.41	401.91	5.23	1.28	2.13
2003	2.80	409.94	1.47	0.040788	1.43	404.02	5.92	1.44	2.11
2004	2.50	412.44	1.49	0.041000	1.44	406.11	6.33	1.53	2.09
2005	2.30	414.74	1.51	0.041210	1.46	408.19	6.55	1.58	2.08
2006	2.30	417.04	1.52	0.041418	1.48	410.24	6.80	1.63	2.05
2007	2.14	419.18	1.54	0.041625	1.49	412.28	6.90	1.64	2.04
2008	2.04	421.22	1.56	0.041830	1.51	414.31	6.91	1.64	2.03
2009	2.01	423.23	1.57	0.042034	1.52	416.32	6.91	1.63	2.01
2010	1.98	425.21	1.59	0.042236	1.54	418.31	6.90	1.62	1.99

## Footnotes

1 For Durkheim individualism is inherent and even contrary to human nature. In fact, in the Durkheimian description an ambivalent human psychological profile emerges, in which the psychological *need not to perish entirely* is however strong and compelling. This belief leads Durkheim to look at suicide as a lost of every reason for existing insofar the individual *alone* is not sufficient end for his activity and he, due his same psychological constitution, cannot live without attachment to some object which transcends and survives him.

2 From a sociological point of view, the study of suicide as a collective phenomenon poses the classic problem of the relationship between structure (macro) and action (micro). In reference to this aspect, for Durkheim the study of suicide rates already provided the opportunity to advance some critique regarding methodological individualism, by affirming the social as an autonomous “sui generis” reality, external and constrictive on individuals. Durkheim had already amply stressed this point in *The Division of Social Work* and in *The Rules of Social Method*. Society is a “sui generis” reality which is substantialized by collective representations which are qualitatively diverse from individual representations and are objectivized by both adaptive cultural elements and material cultural elements. “Social life, so crystalized and fixed by material support finds itself externalized and acts from the outside on us” (Durkheim 1969 [1897], p. 375). Then, there is a whole “fully free collective life” (*cit.* p. 377), currents of opinion, “waves” of optimism and discouragement, “gusts of sadness” “philanthropic appeals”, which despite not being able to condense into objective forms are equally external and constrictive. Therefore, within this framework, suicide as collective phenomenon expresses the constrictive nature of cultural models in orienting our perceptions and actions. This is what Durkheim means when he asserts that social forces are as real as cosmic forces and that the *moral constitution* of the collectivity establishes its “contingent” of voluntary deaths, its *natural inclination* toward suicide.

3 In addition to the problematic question of the relationship between structure (macro) and action (micro), from a sociological point of view the study of suicide as a collective phenomenon poses the classic problem of the relationship between *Nature* and *Nurture* too. In reference to this second aspect, an encouraging support for a *sociologica*l approach toward suicide actually comes from psychiatric and biological fields, which up to World War II assumed suicide as *exclusive* symptom of this or that form of neurotic or psychotic state (Esquirol 1838; De Fleury 1924; Achille-Delmas 1932). Today, findings of numerous empirical studies lead to the conclusion that there is no empirical evidence that suicide is *universally* correlated to identifiable forms of mental disease or pathological depression. They lead us to confirm Durkheim’s conclusion that at the macro level natural, bio-pathological factors are not able to establish a *general law* that can explain suicide rate variability over time, among different categories and social environments, carrying out a role of *predisposition* rather than a role of *precipitation factors,* which seems to be carried out by social causes such as Durkheim believed. For example, today the literature on the relationship between depression and suicide, confirming the lack of systematic correlations between suicide and mental illness (for example, only 10 % of schizophrenics, Saha et al. 2007; Qin 2011), suggests a convergence between psychiatric studies and sociological studies. As regards schizophrenia, for example, the risk of committing suicide seems largely influenced by conditions such as weakened social relationship nets (living alone, singles, separated, divorced and widowed, constitute the vast majority of suicides (i.e. Drake et al. 1984; Harkavy-Friedman et al. 1999; Roy and Draper 2002). On another front, current research still leave the question open as to whether or not there may be an genetic component to suicide independent of a psychiatric disorder. On the one hand, research on twins produced mixed results (Voracek and Loibl 2007. On the other hand, although some studies in neuroscience and neurobiology areas found evidence for a relationship between low serotonin levels (low enzymatic levels of 5-hydroxyindoleacetic acid-5-HIAA) and suicide (the genes that control serotoninergic activity represent ideal “candidate” genes for aggressive and impulsive behaviors which can lead to self harming behaviors; i.e. Anisman et al. 2008), it is still unclear whether the mental illness such as depression determines low levels of serotonin enzyme or *vice versa,* and, therefore, whether these biochemical factors can show an etiological role independent of the psychiatric pathology and can be transmitted hereditarily. Similarly, although research found that density of Von Economo neuron, which are crucial in developing complex emotional stimuli and making the individual emotionally hypersensitive, is much greater in suicides (Brüne et al. 2011), nevertheless these bio-psycho-pathological factors seem to be necessary but not sufficient conditions for definitive predictor value of suicidal behavior. Indeed, they seemed to interact with interpersonal social relations. In this regards, interesting results come from the prevention front too. Research has amply demonstrated that suicide is actually committed when symptoms regress under appropriate pharmacological treatment (Hawton et al. 2005; Pompili et al. 2007) and that social isolation, lack of rewarding relationships with relatives and friends, can seriously undermine the chances of success of any psychotherapeutic and rehabilitative intervention (Montross et al. 2005). In conclusion, one has the impression that *non sociological suicide theories require the intervention of social theorizing*, specifically, in fact, Durkheim’s.

4 In this meaning Durkheim says that the individual paradoxically submits himself to the influence of the collectivity “at the very moment he frees himself from his social environment” (*cit.* p. 264).

5 Referring to religion, Durkheim peremptorily refuted the idea that religion protects people from committing suicide because of theological arguments which condemn the suicidal act but he attributed its prophylactic action on the specific circumstance that it constitutes a “society”. The fact that Protestants committed suicide more than Catholics suggested to Durkheim that the fundamental difference lay in the peculiar structuring of the two religious systems when faced with the problem of free conscience along with the distinct individualistic nature of Protestantism compared to Catholicism. From his perspective Protestantism favored the proliferation of suicidal acts by contemplating free inquiry, emphasizing the autonomy of the individual in matters of faith and, consequently, weakening ties to the reference group. The Protestant is practically “alone in front of God”, invested with a sense of personal responsibility so that he ‘must only count on himself’ and lives his faith outside of an institutionalized dimension. Therefore, Protestant Church appeared to Durkheim essentially less integrated than the Catholic Church. Catholic Church, due to a strong nucleus of dogmas and rites which have to be shared by all the faithful and serve to create ties among every individual believer and the extended confessional group, was to Durkheim a source of identity and integration for the faithful.

6 According to Durkheim the ‘parent’ role was more incisive than the role of spouse in preventing suicide. He presented analytical findings to support these arguments. Our author, for example, showed how having one child actually halved the likelihood of committing suicide and married couples with children and the widowed with children had a preservation coefficient well above that of childless married and widowed persons. The prophylactic action that married people with children enjoyed was directly related to the size of the family. The more offspring, the lower the suicide rate of parents. In other words, according to the author family “density” determined the intensity of interactive relationships and therefore the degree of domestic integration.

7 Under such circumstances, in fact, individual desires, for Durkheim limitless by nature, are set at such a high level of expectation that inevitable disappointments, continuous competition anxiety, perennial states of non-fulfillment are inevitable. Loss of regulating power keeps on moving the bar of expectations always a little higher so that the vainness of a senseless quest, because it never completely satisfies, renders the individual more likely to commit suicide.

8 In times of great economic booms, in fact, it is easy to upset value standards that society uses to regulate needs, fixing the prospects of rewards for each social function for the greater common good. The growth of opportunities, in fact, excites desires, and dulls the conscience which distinguishes what claims and rights/privileges are really legitimate, or on the contrary, what claims are excessive and arbitrary.

9 Finding more constraints than advantages in marriage, women instead compensate for the loss of family protection due to divorce with greater freedom. The different moral constitution between men and women suggested from Durkheim explains his defense of monogamy, which far from being a sacrifice for men is an advantage because it protects men “from sufferings connected to their freedom” (*cit*. p. 334).

10 Durkheim studied the variability of suicide rates by analyzing suicide data in France, Prussia, England, Saxony, Bavaria and Denmark from 1841 to 1872. The author found three variability or change points in the French series of suicide rates connected to 1846, 1860 and 1864; two change points in the Prussian suicide time series in 1849 and 1866; and two in the Saxon suicide data in 1848 and 1865. These changes were *incremental* variations and suggested to Durkheim the idea that suicide trends were *direct* expression of an increasing economic (industrial, financial, commercial, infrastructural…) and cultural modernization process which involved France, Prussia etc. in the same years.

11 For Halbwachs the *Convergence Law* is effect of the modernization process A measure of degree of convergence among suicide rates in more industrialized nations and in developing nations, the *coefficient of dispersion,* allowed Halbwachs to corroborate the validity of his hypothesis. More recently, by analyzing the suicide rates in Italy, Condorelli (1998) supported Halbwachs’ *Convergence Law.* After controlling for autocorrelations, from 1864 to 1995 an increase in *convergence,* that is, a reduction in dispersion coefficients of suicide rates among Northern Italian regions and Southern Italian regions was found. The analysis showed a reduction in rise of suicide rates relating to the *industrial triangle regions* (Piedmont, Lombardy, Liguria), the first regions to be transformed by the industrialization process, and a constant increase of suicide growth rate in the Central and Southern Italian regions, such as Umbria, Marche, Abruzzo-Molise, Basilicata, Puglia, Calabria, which were considered involved in a more recent development of industrialization-modernization process.

12 From 1995 to 2010, Italian Institute of Statistics provides suicide data disaggregated by causes or reason of suicide. As the following table shows, after 2007 suicides due to economic reasons linked to recession tend to increase compared to suicides due to non-economic reason (*ISTAT*2012a, b):

13 *Emergence*, *novelty*, *surprise* are the words used to describe the new patterns of relationship, the new high-level properties of the system generated by collective dynamics of its components or by *nonlinear**interactions* among its constituent parts (Holland 1998; McDaniel and Driebe 2010).

14 Considering the case of discrete generations, in Malthusian model for each element in population t there will be *a* elements in population t + 1 (time interval of length 1 (Δt = 1)). The corresponding finite-difference equation is

$${\text{N}}_{\text{t + 1}} - {\text{N}}_{\text{t}} = a{\text{N}}_{\text{t}}$$

and

$${\text{N}}_{\text{t + 1}} = {\text{N}}_{\text{t}} + a{\text{N}}_{\text{t}}$$

or else

$${\text{N}}_{\text{t + 1}} = b{\text{N}}_{\text{t}}$$

where

$$b = 1 + a$$

(if the growth rate *a* is 100 %, then we have: N_t+1_ − N_t_ = 100 % N_t_, and N_t+1_ = N_t_ + 100 % N_t_. Obviously, it means that N_t+1_ is the double of N_t_, that is, N_t+1_ = 2 N_t_, *b* = 2: N_t+1_ repeats N_t_ and adds the 100 % of N_t_. Therefore *b* is equal at 1 + *a*, and in turn is equal to the value of Anti-logarithm of *e*^*a*^). Starting from initial condition N_0_ and being given the equation N_t+1_ = *b*N_t_, the solution is obtained by iteration or by a general law, that is:

$$\begin{aligned} &{\text{N}}_{1} = b{\text{N}}_{ 0} \hfill \\ &{\text{N}}_{2} = b{\text{N}}_{1} = b\left({b{\text{N}}_{0}} \right) = b^{2} {\text{N}}_{0} \hfill \\ &{\text{N}}_{3} = b{\text{N}}_{2} = bb\left({b{\text{N}}_{0}} \right) = b^{3} {\text{N}}_{0} \hfill \\ &{\text{N}}_{4} = b{\text{N}}_{3} = b\left({b\left({b\left({b{\text{N}}_{0}} \right)} \right)} \right) = b^{4} {\text{N}}_{0} \hfill \\ & \ldots\ldots\ldots\ldots\ldots\ldots\ldots\ldots\ldots \hfill \\ &{\mathbf{N}}_{{\mathbf{t}}} = {\mathbf{N}}_{{\mathbf{0}}} b^{{\mathbf{t}}} \hfill \\ \end{aligned}$$

*Example* N_t+1_ is the double of N_t_

$${N}_{{{t} + {1}}} = {2N}_{t},$$

$${N}_{0} = 1,{b} = 2.$$

By iteration, we have:

$$\begin{aligned} {\text{N}}_{ 1} &= 2{\text{N}}_{ 0} \hfill \\ {\text{N}}_{ 2} &= 2{\text{N}}_{ 1} = 2^{2} {\text{N}}_{0} \hfill \\ N_{3}& = 2N_{2} = 2^{3} N_{0} \hfill \\ {\text{N}}_{4}& = 2{\text{N}}_{ 3} = 2^{4} {\text{N}}_{ 0} \hfill \\ \end{aligned}$$

or, by general law,

$$N_{t} = 2^{t} N_{0}$$

15 Attractors are geometric structures described by the system in its evolution. These geometric shapes attract all the orbits starting from different initial conditions, hence the name attractors. These shapes can be discovered by using *state space or phase space mapping.* A phase space represents a graphic backdrop for presenting the motion of time-based data (Kiel and Elliott 1997: 27). We construct a XY graph and place on the horizontal axis or abscissa the observed values x_t_ and on the vertical axis or ordinates the same values measured with time delays or x_t−n_ delayed by 1, 2,…3, and so on time lags. The most suitable time lag for reconstructing the attractor is found by experiments. Usually, however, it is set at equal 1. The values x_t_ and x_t−n_ constitute the coordinates of a point on a multidimensional space, and the set of points identified in this way describe an orbit which in the long run traces the typical attractor of the observed system. In fact, according to Takens theorem (1980) the geometric shape thereby constructed is topologically equivalent to the original attractor, that is the one that would be reconstructed if we knew exactly the number and the values of variables explaining the observed dynamical process (or if we knew the *governing equation**of motion* of the nonlinear system). In fact, it maintains the properties of the original attractor, including the dimension. This topological equivalence is called by a complicated term *diffeomorphism.* Every system has its own attractor, with its typical shape. For example, if a system is stable, (forced pendulum), the attractor is at a fixed point. If the system display periodic behavior the attractor is a limit cycle (free pendulum). When the system is chaotic, the attractor assumes the strangest shapes, thus the attractor is called *strange*. In the case of logistic equations, the system’s attractor is reconstructed considering a bi-dimensional space x_t_/x_t−1_. Setting the x_t_ values on the abscissa and x_t-1_ values on the ordinate, the points that are identified by the coordinates x_t_ and x_t−1_ form a hill-shaped attractor.

16 Standardization allows us to make comparable identical variables belonging to different distributions, or else variables which are expressed in different units of measurement. It is useful in our case: unlike target data values, logistic values are between 0 and 1.

17 Suicide data reported during Fascism could be even under-represented: it is plausible to think that suicide cases could have been concealed and classified in different causes of death, insofar suicide was considered a shameful deviance and a dangerous indicator of weakness of the “new man ideology” whom Fascism advocated.

18 In 1862 198,666 marriages were celebrated.Throughout the last century the number of marriages grew in absolute value until to stabilize at 250,000 per year; however marriage rates per 1000 inhabitants drastically decreased from 8.2 in 1862 to 3.8 in 2009. There were just 717 marital separations in 1881. Already in 1921 they doubled and increased more and more in decades later: about 5000 to late forties, 10,000 in 1970, 25,000 in 1978, 50,000 in 1994, until to get to 84,165 in 2008.

## References

[CR1] Achille-Delmas F (1932). Psycologie pathologique du suicide.

[CR2] Agerbo E, Stack S, Petersen L (2011). Social integration and suicide: Denmark, 1906–2006. Soc Sci J.

[CR3] Anderson P (1972). More is different. Science.

[CR4] Anisman H, Du L, Palkovits M (2008). Serotonine receptor subtype and p11 mRNA expression in stress-relevant brain regions of suicide and control subjects. J Psychiatry Neurosci.

[CR5] Bailey K (1984). Beyond functionalism: toward a nonequilibrium analysis of complex social systems. Br J Sociol.

[CR6] Bak P, Chen K (1991). Self-organized criticality. Sci Am.

[CR7] Ball P (2012). Why society is a complex matter.

[CR8] Bauman Z (1995). Life in fragments. Essays in postmodern morality.

[CR9] Bauman Z (1997). Postmodernity and its discontents.

[CR10] Bauman Z (2000). Liquid modernity.

[CR11] Beck U, Beck E (1994). Gernsheim: Riskante Freiheiten: Individualisierung in modernen Gesellschaften.

[CR12] Bertuglia CS, Vaio F (2003). Nonlinerità, Caos, Complessità.

[CR13] Blakely TA, Collings SCD, Atkinson J (2003). Unemployment and suicide. Evidence for a casual association?. J Epidemiol Community Health.

[CR14] Blasco-Fontesilla H (2012). Worldwide impact of economic cycles on suicide trends over 3 decades: differences according to level of development. A mixed effect model study. BMJ Open.

[CR15] Braun M (1993). Differential equations and their applications.

[CR16] Breault KD (1986). Suicide in America: a test of Durkheim’s theory of religious and family integration. Am J Sociol.

[CR17] Breault KD, Barkey K (1982). A comparative analysis of Durkheim’s theory of egoistic suicide. Sociol Q.

[CR18] Breault KD, Kposowa AJ, Pickering WSF, Walford G (2000). Social integration and marital status. A multivariate individual-level study of 30,157 suicides. Durkheim’s suicide: a century of research and debate.

[CR19] Brown C (1991). Ballots of tumult. A portrait of volatility in American voting.

[CR20] Brüne M, Schöbel A, Karau R, Faustmann PM, Dermietzel R (2011). Neuroanatomical correlates of suicide in psychosis: the possible role of von Economo neurons. PLoS One.

[CR21] Campbell DK, Mayer-Kress G, Grebogi C, Yorke JA (1997). Chaos and politics: applications of nonlinear dynamics to socio-political issues. The impact of chaos on science and society.

[CR22] Condorelli R (1998). Al fondo dell’abisso. Un approccio bayesiano alla fenomenologia del suicidio.

[CR23] Condorelli R (2007). Complessità e Controllo sociale.

[CR24] Condorelli R (2013). A Bayesian analysis of suicide data. Testing the Durkheim’s suicide theory: a suicide study in Italy. Qual Quan.

[CR25] Condorelli R, Tchuenche JM (2013). Complexity and Chaos theory in social sciences: scientific philosophy or quantitative science?. Dynamical systems: theory, applications, and future directions.

[CR26] Condorelli R (2013). Applied nonlinear dynamical system in social science. A nonlinear model for social control system: an application to Italian coercion system. Qual Quan.

[CR27] Cutright P, Stack S, Fernquist R (2007). Marital status integration, suicide disapproval, and societal integration as explanations of marital status differences in female age-specific suicide rates. Suicide Life Threat Behav.

[CR28] De Fleury M (1924). L’angoisse humaine.

[CR29] De Rosa L (1980). La rivoluzione industriale in Italia.

[CR30] De Vogli R, Marmot M, Stuckler D (2012). Letter: excess suicides and attempted suicides in Italy attributable to the great recession. J Epidemiol Community Health.

[CR31] De Vogli R, Marmot M, Stuckler D (2013). Strong evidence that the economic crisis caused a rise in suicides in Europe: the need for social protection. J Epidemiol Community Health.

[CR32] Dendrinos D (1992). The dynamics of cities.

[CR33] Dooley K, Hamilton P, Cherri M, West B, Fisher P, Eve RA, Horsfall S, Lee ME (1997). Chaotic behavior in society. Adolescent childbearing in Texas, 1964–1990. Chaos, complexity, and sociology. Myths, models and theories.

[CR34] Drake R, Gates C, Cotton P, Whittaker A (1984). Suicide among schizophrenics. Who is at risk?. J Nerv Mental Dis.

[CR35] Durkheim É (1897) Le suicide: étude de sociologie. Alcan, Paris (transl. Il suicidio e l’educazione morale. Torino, UTET, 1969)

[CR36] Elaydi SN (1991). An introduction to difference equations.

[CR37] Elliott E, Kiel DL, Kiel DL, Elliott E (1997). Introduction. Chaos theory in the social science: foundations and applications.

[CR38] Esquirol JE (1838). Des maladies mentales considérées sous les rapports médical, hygiénique et médico-legal.

[CR39] EURES (2012). Il suicidio in Italia al tempo della crisi—II Rapporto EURES.

[CR40] Feigenbaum M (1978). Quantitative universality for a class of non-linear transformations. J Stat Phys.

[CR41] Fernquist RM (2009). Suicide rates and status integration in America. Arch Suicide Res.

[CR42] Gibbs JP, Martin WT (1964). Status integration and suicide. A sociological study.

[CR43] Gleick J (1987). Chaos: making a new science.

[CR44] Gottman JM, Murray JD, Swanson CC, Tyson R, Swanson KR (2002). The mathematics of marriage. Dynamic nonlinear models.

[CR45] Granados JA (2005). Increasing mortality during the expansions of the US economy, 1900–1996. Int J Epidemiol.

[CR46] Grossmann S, Mayer-Kress G (1989). Chaos in the international arms race. Nature.

[CR47] Guastello SJ, Guastello DD (2008). Dynamics of attitudes and genetic processes. Nonlinear Dyn Psychol Life Sci.

[CR48] Halbwachs M (1930). Les causes du suicide.

[CR49] Harkavy-Friedman JM, Restifo K, Malaspina D (1999). Suicidal behavior in schizophrenia: characteristics of individuals who had and had not attempted suicide. Am J Psychiatry.

[CR50] Harvey DL, Reed M, Kiel DL, Elliott E (1997). Social Sciences as the Study of Complex Systems. Chaos theory in the social science: foundations and applications.

[CR51] Hawton K, Sutton L, Haw C (2005). Schizophrenia and suicide: systematic review of risk factors. Br J Psychiatry.

[CR52] Holland JH (1998). Emergence: from Chaos to order.

[CR53] Hood-Williams J (1996). Studying suicide. Health Place.

[CR54] Horgan J (1996). The end of science: facing the limits of knowledge in twilight of the scientific age.

[CR55] Huckfeldt R (1989). Noncompliance and the limits of coercion: the problematic enforcement of unpopular laws. Math Comput Model.

[CR56] Huckfeldt R (1990). Structure, indeterminacy and chaos. A case for sociological law. J Theor Polit.

[CR57] Innamorati M, Tamburello A, Lester D, Amore M, Girardi P, Tatarelli R, Pompili M (2010). Inequalities in suicide rates in the European Union’s elderly: trends and impact of macro-socioeconomic factors between 1980 and 2006. Can J Psychiatry.

[CR58] ISTAT (2012). I suicidi in Italia: tendenze e confronti, come usare le statistiche.

[CR59] ISTAT (2012 and previous years from 1864) Suicidi e Tentativi di Suicidio in Italia. Istituto Nazionale di Statistica, Roma

[CR60] Kaplan D, Glass L (1995). Understanding nonlinear dynamics.

[CR61] Kauffman S (1995). At home in the universe: the search for laws of self-organization and complexity.

[CR62] Kostelich EJ, Armbruster D (1996). Introductory differential equations. From linearity to chaos.

[CR63] Kposowa A (2003). Divorce and suicide risk. J Epidemiol Community Health.

[CR64] Krujits CS (1977). The suicide rate in the western world since world war II. Neth J Sociol.

[CR65] Langton CG (1990). Computation at the edge of chaos. Phys D.

[CR66] Lester D (2006). Islam ad suicide. Arch Suicide Res.

[CR67] Li T, Yorke JA (1975). Period three implies chaos. Am Math Mon.

[CR68] Lorent V, Kunst AE, Huisman M, Bopp M, Mackenbach J (2005). A European comparative study of marital status and socio-economic inequalities in suicide. Soc Sci Med.

[CR69] Luhmann N (1984). Soziale systeme.

[CR70] Luhmann N, Geyger F, van der Zowen J (1986). The autopoiesis of social systems. Sociocybernetics paradoxe.

[CR71] Luo F, Florence CS, Quispe-Agnoli M, Ouyang L, Crosby AE (2011). Impact of business cycles on US suicide rates, 1928–2007. Am J Public Health.

[CR72] Marion R (1999). The edge of organization.

[CR73] Maturana H, Varela F (1984). El arbol del conocimiento.

[CR74] May RM (1976). Simple mathematical models with very complicated dynamics. Nature.

[CR75] McDaniel RR, Driebe DJ (2010). Uncertainty and surprise in complex systems.

[CR76] Miller JH, Page SE (2007). Complex adaptive systems. An Introduction to computational models of social life.

[CR77] Montross LP, Zisook S, Kasckow J (2005). Suicide among patients with schizophrenia: a consideration of risk and protective factors. Ann Clin Psychiatry.

[CR78] Parsons T, Shils EA (1951). Toward a general theory of action.

[CR79] Pescosolido B (1990). The social context of religious integration and suicide. Sociol Q.

[CR80] Pescosolido BA, Georgianna S (1989). Durkheim, suicide, and religion: toward a network theory of suicide. Am Sociol Rev.

[CR81] Pompili M, Amador XF, Girardi P (2007). Suicide risk in schizophrenia: learning from the past to change the future. Ann Gen Psychiatry.

[CR82] Priesmeyer RH, Albert A (1995). Logistic regression: a method for describing, interpreting, and forecasting social phenomenon with nonlinear equations. Chaos and society.

[CR83] Prigogine I (1997). The end of certainty.

[CR84] Prigogine I, Nicolis G (1977). Self-organization in non-equilibrium systems.

[CR85] Prigogine I, Stengers I (1979). La nouvelle alliance. Metamorphose de la science.

[CR86] Prigogine I, Stengers I (1984). Order out of chaos: man’s new dialogue with nature.

[CR87] Qin P (2011). The impact of psychiatric illness on suicide: differences by diagnosis of disorders and by sex and age of subjects. J Psychiatr Res.

[CR88] Reeves A, Stuckler D, McKee M, Gunnell D, Chang SS, Basu S (2012). Increase in state suicide rates in the USA during economic recession. Lancet.

[CR89] Rendall MS, Weden MM, Favreault MM, Waldron H (2011). The protective effect of marriage for survival: a review and update. Demography.

[CR90] Roy A, Draper R, De Leo D, Schmidtke A, Diekstra RFW (2002). Suicide among Psychiatric Hospital Inpatients. Suicide prevention.

[CR91] Saha S, Chant D, McGrath J (2007). A systematic review of mortality in schizophrenia: is the differential mortality gap worsening over time?. Arch Gen Psychiatry.

[CR92] Saperstein AM (1984). Chaos—a model toward for the outbreak of war. Nature.

[CR93] Saperstein AM, Kiel DL, Elliott E (1997). The prediction of unpredictability: applications of the new paradigm of chaos in dynamical systems to the old problem of the stability of a system of hostile nations. Chaos theory in the social science: foundations and applications.

[CR94] Saperstein AM, Eve RA, Horsfall S, Lee ME (1997). The origin of order and disorder in physical and social deterministic systems. Chaos, complexity, and sociology. Myths, models and theories.

[CR95] Simpson ME, Conklin GH (1989). Socioeconomic development, suicide and religion: a test of Durkheim’s theory of religion and suicide. Soc Forces.

[CR96] Stack S (1983). The effect of the decline in instituzionalized religion on suicide. J Sci Study Relig.

[CR97] Stack S (1985). The effect of domestic/religious individualism on suicide, 1954–1978. J Marriage Fam.

[CR98] Stack S (1989). The impact of divorce on suicide in Norway, 1950–1980. J Marriage Fam.

[CR99] Stack S (1990). The effect of divorce on suicide in Denmark 1951–1980. Sociol Q.

[CR100] Stack S (1992). The effect of divorce on suicide in Finland: a time series analysis. J Marriage Fam.

[CR101] Stack S (1993). The effect of modernization on suicide in Finland 1800–1984. Sociol Perspect.

[CR102] Stack S (2000). Suicide: a 15 year review of the sociological literature. Part II: modernization and social integration perspectives. Suicide Life Threat Behav.

[CR103] Stack S, Kposowa AJ (2011). Religion and suicide acceptability: a cross-national analysis. J Sci Study Relig.

[CR104] Stack S, Scourfielf J (2013). Recency of divorce, depression, and suicide risk. J Fam Issues.

[CR105] Stewart I (1989). Does God play dice? The mathematics of chaos.

[CR106] Thomas K, Gunnell D (2010). Suicide in England and Wales 1861–2007: a time-trends analysis. Int J Epidemiol.

[CR107] Tsebelis G, Sprague J (1989). Coercion e revolution: variations on a predator-prey model. Math Comput Model.

[CR108] Tsebelis G, Sprague J (2010). Coercion e revolution: variations on a predator-prey model and computer disorder?. Criminol Pub Policy.

[CR109] von Bertalanffy L (1969). General system theory.

[CR110] Voracek M, Loibl LM (2007). Genetics of suicide: a systematic review of twin studies. Wien Klin Wochenschr.

[CR111] Waldrop M (1992). Complexity: the emerging science at the edge of order and chaos.

[CR112] West BJ (1997). Chaos and related things: a tutorial. J Mind Behav.

[CR113] Wray M, Colen C, Pescosolido B (2011). The sociology of suicide. Annu Rev Sociol.

[CR114] Wyder M, Ward P, De Leo D (2009). Separation as a suicide risk factor. J Affect Disord.

[CR115] Ying YH, Chang K (2009). A study of suicide and socioeconomic factors. Suicide Life Threat Behav.

